# Critical Assessment of Protein Cross-Linking and Molecular Docking: An Updated Model for the Interaction Between Photosystem II and Psb27

**DOI:** 10.3389/fpls.2016.00157

**Published:** 2016-02-18

**Authors:** Kai U. Cormann, Madeline Möller, Marc M. Nowaczyk

**Affiliations:** Plant Biochemistry, Ruhr University BochumBochum, Germany

**Keywords:** cyanobacteria, photosystem II, assembly, CX-MS, SPR, Psb27

## Abstract

Photosystem II (PSII) is a large membrane-protein complex composed of about 20 subunits and various cofactors, which mediates the light-driven oxidation of water and reduction of plastoquinone, and is part of the photosynthetic electron transfer chain that is localized in the thylakoid membrane of cyanobacteria, algae, and plants. The stepwise assembly of PSII is guided and facilitated by numerous auxiliary proteins that play specific roles in this spatiotemporal process. Psb27, a small protein localized in the thylakoid lumen, appears to associate with an intermediate PSII complex that is involved in assembly of the Mn_4_CaO_5_ cluster. Its precise binding position on the PSII intermediate remains elusive, as previous approaches to the localization of Psb27 on PSII have yielded contradictory results. This was our motivation for a critical assessment of previously used methods and the development of an improved analysis pipeline. The combination of chemical cross-linking and mass spectrometry (CX-MS) with isotope-coded cross-linkers was refined and validated with reference to the PSII crystal structure. Psb27 was localized on the PSII surface adjacent to the large lumenal domain of CP43 on the basis of a cross-link connecting Psb27-K91 to CP43-K381. Additional contacts associating Psb27 with CP47 and the C-termini of D1 and D2 were detected by surface plasmon resonance (SPR) spectroscopy. This information was used to model the binding of Psb27 to the PSII surface in a region that is occupied by PsbV in the mature complex.

## Introduction

Photosystem II (PSII) catalyzes the light-driven oxidation of water (McEvoy and Brudvig, 2006; Cox et al., 2013). It is the only known enzymatic system that is able to use water as a universal source of electrons for the reduction of plastoquinone, the initial reaction of photosynthetic electron transport (PET). PSII is a large membrane-protein complex that is localized in the thylakoid membrane of all organisms that perform oxygenic photosynthesis, including cyanobacteria, algae, and plants. Cyanobacterial PSII has been successfully characterized by X-ray crystallography, and detailed structural models with a resolution down to 1.9 Å are available (Ferreira et al., 2004; Guskov et al., 2009; Umena et al., 2011). These PSII structures reveal the spatial disposition of up to 20 protein subunits and multiple cofactors, such as chlorophylls, carotenoids, and lipids. In particular, a comprehensive analysis of the water-oxidizing complex (WOC), which is composed of an inorganic Mn_4_O_5_Ca cluster and its coordinating amino acid residues, has been facilitated by the atomic level of resolution of the latest structures (Umena et al., 2011; Suga et al., 2015). The core complex is a heterodimer of the D1 and D2 subunits (PsbA and PsbD), which coordinate most of the cofactors involved in electron transfer. Two domains of the adjacent light-harvesting proteins CP43 and CP47 protrude out of the plane of the membrane into the thylakoid lumen, and act as anchoring points for the soluble extrinsic subunits PsbO, PsbV, and PsbU, which shield the WOC on the lumenal face of the thylakoid membrane (Roose et al., 2007). The remaining 13 small (<10 kDa) and hydrophobic (with 1–2 transmembrane helices) subunits play various roles during PSII assembly and in the mature complex, but the exact functions of many of them remain obscure (Shi et al., 2012). However, assembly of the different components during PSII biogenesis is an intricate process that is coordinated by a network of auxiliary proteins (Mulo et al., 2008; Nixon et al., 2010; Becker et al., 2011; Nickelsen and Rengstl, 2013). These accessory factors direct the spatiotemporal formation and distribution of precursor complexes in a coordinated assembly process, including a series of distinct, metastable assembly intermediates. Structural analysis of these intermediate PSII complexes is severely hampered by their low abundance and transient nature, with the consequence that none of these complexes has been successfully analyzed by X-ray crystallography so far.

One of the best studied proteins involved in PSII biogenesis is the auxiliary factor Psb27 (Nowaczyk et al., 2010; Fagerlund and Eaton-Rye, 2011; Mabbitt et al., 2014), which forms part of several distinct PSII assembly intermediates and plays a role in both PSII biogenesis and repair (Nowaczyk et al., 2006; Roose and Pakrasi, 2008; Grasse et al., 2011; Liu et al., 2011b; Komenda et al., 2012).

Psb27 has been shown to stabilize unassembled CP43 in a precomplex that is composed of the membrane-embedded subunits CP43, PsbK, PsbZ, and Psb30 (Komenda et al., 2012). After formation of a stable (inactive) monomeric PSII intermediate including Psb27, but not the extrinsic proteins PsbO, PsbV, and PsbU, it might facilitate assembly of the WOC, as this step is followed by the release of Psb27, photoactivation of the cluster and association of the extrinsic proteins (Roose and Pakrasi, 2008). In cyanobacteria, Psb27 is modified by an N-terminal lipid modification and attached to the lumenal side of the thylakoid membrane (Nowaczyk et al., 2006). Moreover, it is involved in acclimation to fluctuating light conditions in higher plants (Hou et al., 2015). Notably, isolation of the monomeric PSII-Psb27 complex revealed species- or preparation-dependent differences. Whereas a complex isolated from *Thermosynechococcus elongatus* via two-step chromatography is devoid of all three extrinsic proteins (Nowaczyk et al., 2006), a similar complex isolated from *Synechocystis* sp. PCC6803 via one-step chromatography still contains considerable amounts of PsbO, but not PsbV or PsbU (Liu et al., 2011b).

The structure of recombinant Psb27 has been solved by NMR spectroscopy (Cormann et al., 2009; Mabbitt et al., 2009) and by X-ray crystallography (Michoux et al., 2012), which reveal a four-helix bundle with a dipolar charge distribution. Part of the surface that is formed by helices III and IV is highly conserved and might facilitate binding to PSII. Several approaches have been used in attempts to localize the Psb27 binding site on the lumenal surface of PSII. *In silico* docking experiments suggested a location for Psb27 that partly overlaps the PsbO binding site (Cormann et al., 2009; Fagerlund and Eaton-Rye, 2011), whereas experimental results based on mass spectrometry (Liu et al., 2011a, 2013) support the localization of Psb27 on the opposite side of CP43 Loop E, pointing toward transmembrane helices III and IV. These conflicting results may be attributed to limitations of the methods used, and this motivated us to undertake a critical assessment of both. We made use of the high-resolution PSII crystal structure for validation of the methods and to define critical parameters for the evaluation of mass spectrometry (MS) data. An optimized workflow involving a combination of isotope-coded cross-linking and MS based on a previous study (Rexroth et al., 2014), as well as surface plasmon resonance (SPR)-based mapping (Cormann et al., 2014) pinpointed a Psb27 binding position in the vicinity of CP43 at the PsbV binding site on the lumenal PSII surface.

## Results

### Identification of Correct Models Derived by Ab Initio Docking Requires Experimental Data

To check the ability of *ab initio* docking algorithms to predict the correct position of soluble subunits at the PSII surface, we made use of the CAPRI protocol for ‘Critical Assessment of PRedicted Interactions’ (Janin et al., 2003). In this method, the three-dimensional structures of free interaction partners are used to model the configuration of the complex of interest, and the results are compared to the experimentally derived coordinates of the assembled proteins. The fraction of native or non-native contacts discovered and the root mean square deviations (RMSD) between the predicted and the experimentally determined positions serve as criteria for classifying the quality of the models obtained as high, medium, acceptable, or incorrect. In the case of PSII, PsbV is the only soluble subunit whose structure is available in both unbound (Kerfeld et al., 2003) and bound (Umena et al., 2011) forms. As no PSII crystal structure without the extrinsic subunits PsbO, PsbV, and PsbU is available, we removed them from the PDB file and docked the unbound PsbV structure to the membrane-intrinsic portion of PSII. This structure served as the reference standard for the following analysis.

**FIGURE 1:**
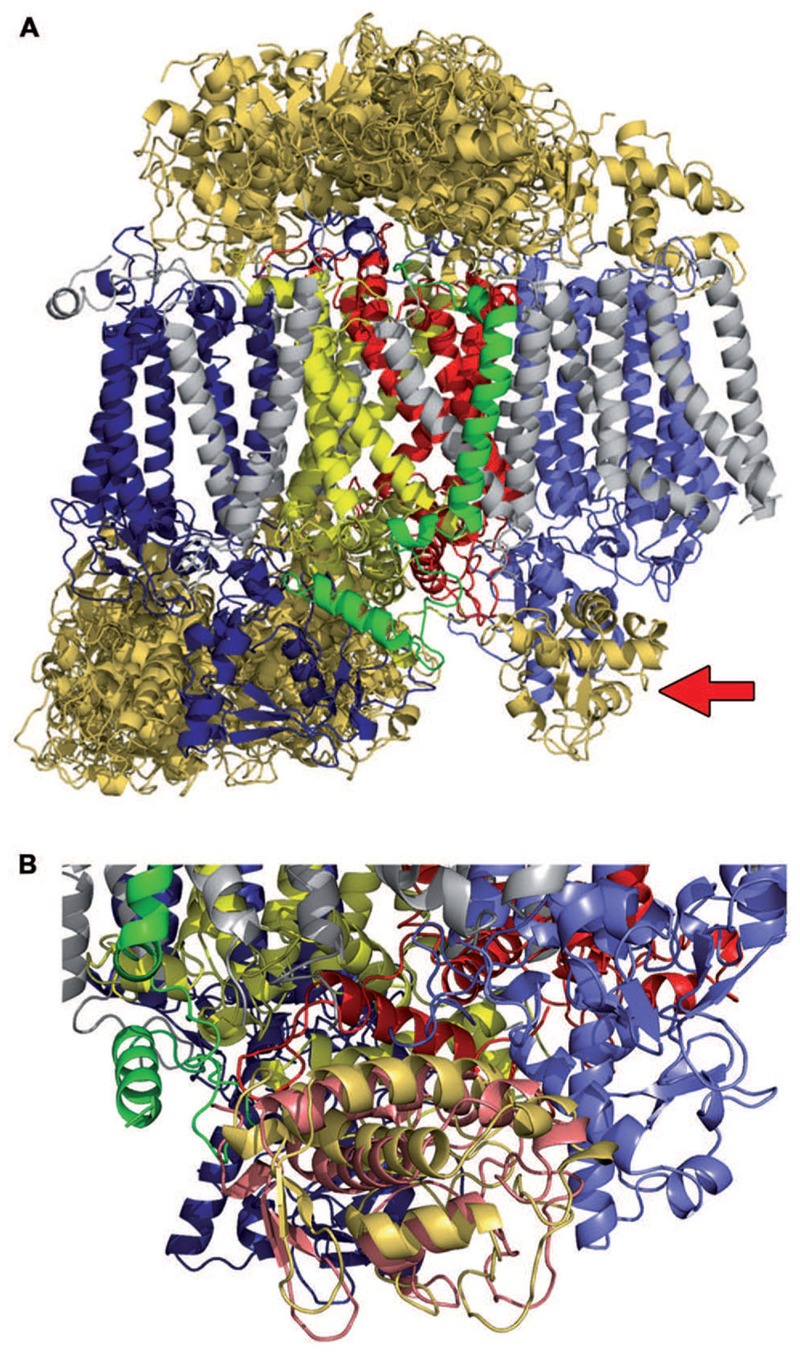
***Ab initio* docking of PsbV (orange) to monomeric Photosystem II using ClusPro server 2.0. (A)** Summary representation of 29 models that were generated on the basis of monomeric PSII without the extrinsic subunits PsbU, PsbO, PsbV (PDB: 3ARC) and the crystal structure of free PsbV (PDB: 1MZ4): 15 models show PsbV on the cytoplasmic side, 14 on the lumenal side. The calculation is based on van der Waals forces and electrostatic interactions. Only one model (red arrow) essentially reproduces the native position of PsbV, but it was ranked 24th of the 29 structures predicted by ClusPro 2.0. **(B)** Overlay of the native (salmon) and modeled (yellow) PsbV binding positions.

The ClusPro server 2.0 (Comeau et al., 2004a,b; Kozakov et al., 2006), which has been employed in most modeling studies due to its top ranking performance in the latest comparative docking experiments (Kozakov et al., 2013; Lensink and Wodak, 2013), distinguishes between four subgroups of PSII models that differ in the energy parameters for contacts, based on hydrophobicity, charge or shape. Whereas three subgroups place PsbV in clearly erroneous positions with contacts to the membrane-embedded residues of PSII, PsbV is exclusively localized at the solvent-accessible interfaces in models favoring interactions based on charge and shape. Within this second group of 29 models, 15 place PsbV on the cytoplasmic side of PSII and 14 assign it to the lumenal side (**Figure 1A**). One of the latter models closely approximates the native position of PsbV adjacent to the CP43 and D1 subunits (**Figure 1B**). With a calculated fraction of 60% of the native interaction site and an RMSD of 5.2 Å relative to its position in the PSII crystal structure, this model is classified as acceptable. However, the software ranks it in position 24 out of 29, based on its cluster size, and thus obviously incorrect models are suggested to represent the native structure. In light of such limitations, only experimental data can distinguish the correct model from the majority of false-positive results.

### Reliable Assignment of Cross-Linked Peptides is Facilitated by Isotopic Labeling

The analysis of cross-linking MS (CX-MS) data is especially challenging owing to the fact that structurally meaningful interpeptide cross-links are obscured by the vastly greater number of non-informative intrapeptide cross-links, dead-end cross-links, and free peptides. Moreover, mass spectrometric analysis of PSII samples is even more difficult, as this protein complex consists of around 20, mostly hydrophobic, subunits per monomer. However, the major problem lies in the overwhelming number of theoretically possible peptides, in particular if post-translational modifications other than the cross-link itself are included in the analysis. Furthermore, the experimental data set may include spectra of copurifying impurities, which are not included in the database used for CX-MS analyses. Hence, careful inspection of the results for false-positive MS2 assignments is mandatory. Thus, we used the high-resolution crystal structure of PSII (Umena et al., 2011) to test and validate our CX-MS approach.

Two PSII samples (inactive, monomeric Psb27-PSII and active, dimeric PSII) were incubated with the isotope-coded cross-linker bis(sulfosuccinimidyl)suberate (BS3, spacer length: 11.4 Å), then digested with trypsin and analyzed by LC-ESI tandem mass spectrometry. Data analysis was performed with StavroX v. 3.1.19 (Götze et al., 2012).

As the false discovery rate represents one of the main difficulties in the identification of cross-linked peptides, most software tools provide a scoring function that estimates the probability of a false positive assignment. StavroX calculates a score based on the identified fragment ions for each precursor mass (Götze et al., 2012). To establish a correlation between the score and the false discovery rate, the dataset is searched analogously against a decoy database consisting of reversed protein sequences. Previous validation of the software based on model systems yielded a false discovery rate of 2% for scores >100 (Götze et al., 2012).

**FIGURE 2:**
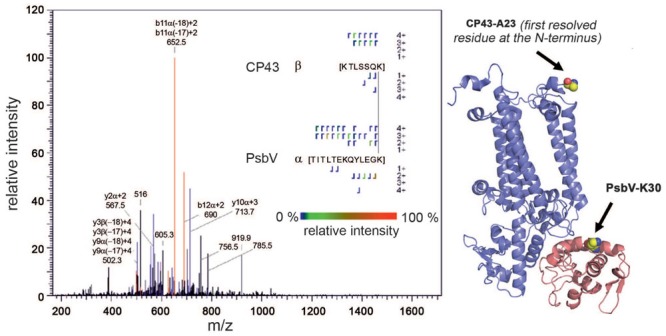
**Example of the identification of false-positive cross-links by StavroX.** Identified b-ions (red), y-ions (blue), and unidentified signals (black) are indicated in the spectrum. Purple signals represent either b- or y-ions. A cross-link between the N-terminal part of CP43 and PsbV was identified by the software (Score: 137), although the domains are located on different sides of the membrane. Furthermore, trypsin should not recognize a BS3-modified lysine.

We first attempted to identify only H_12_-BS3 cross-links in the two PSII data sets, in order to test the reliability of the software. When scores ≤100 were considered, the number of hits in the decoy database equaled the number of possible cross-linked peptides identified with the native database. For scores >100 the number of peptide identifications for the native sequences clearly exceeded that for the decoy database, but StavoX still reported 9 and 10 decoy hits compared to 65 and 80 hits for the native database in the two datasets, respectively. In addition, the results obtained with the native database contained false-positive discoveries with scores >100, although these “cross-links” clearly contradict the PSII crystal structure. Thus, despite the fact that it is based on nearly complete assignment of the entire fragment ion series, the result shown in **Figure 2** suggests that the N-terminal portion of CP43 is cross-linked to PsbV, although these sequences are located on different sides of the membrane. Even though the number of false discoveries is clearly lower than that of true hits, it might nevertheless account for the contradictory and artefactual structural models, since each suggested assignment is the basis for a structural constraint.

**FIGURE 3:**
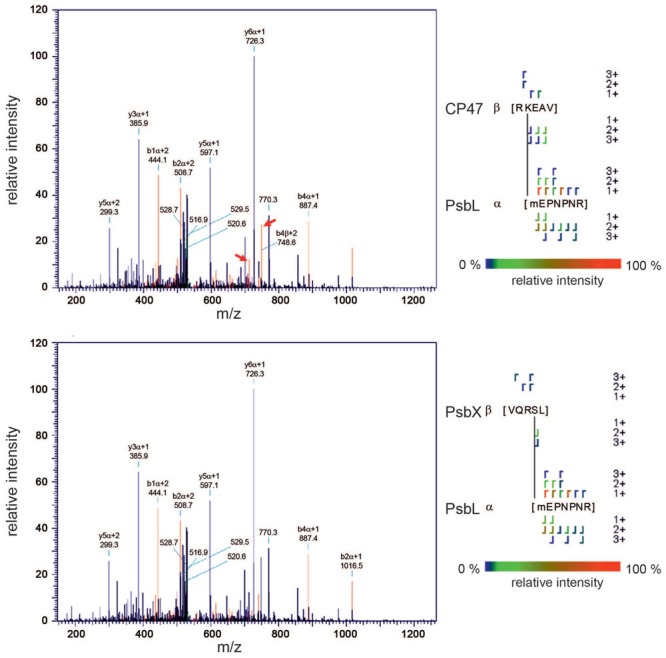
**Example of an ambiguous assignment.** MS2 fragmentation pattern that was either assigned to H_12_-BS3 cross-linked peptides of PsbL and CP47 (upper spectrum, Score: 136) or PsbL and PsbX (lower spectrum, Score: 100). In the case of PsbL and CP47 only two (b-)ions were assigned to the β-peptide (red arrows), whereas for PsbX no specific ions were found.

Moreover, assignments that are based only on the identification of H_12_-BS3 cross-linked peptides might be ambiguous, even though the score calculated for each identification is above the threshold. **Figure 3** shows an MS2 fragment pattern that was assigned to cross-linked peptides of PsbL and CP47 (score 136) and PsbL and PsbX (score 100). Most of the observed fragments were assigned to the α-peptide (PsbL), whereas the identity of the β-peptide is ambiguous. In the upper spectrum only two (b-)ions of the β-peptide (CP47, red arrows) were found, and in the lower spectrum none of the PsbX-specific fragments were identified. Although the identification of the PsbL/CP47 cross-link is more reasonable – also from the structural perspective – there is still considerable uncertainty about the correct assignment.

**FIGURE 4:**
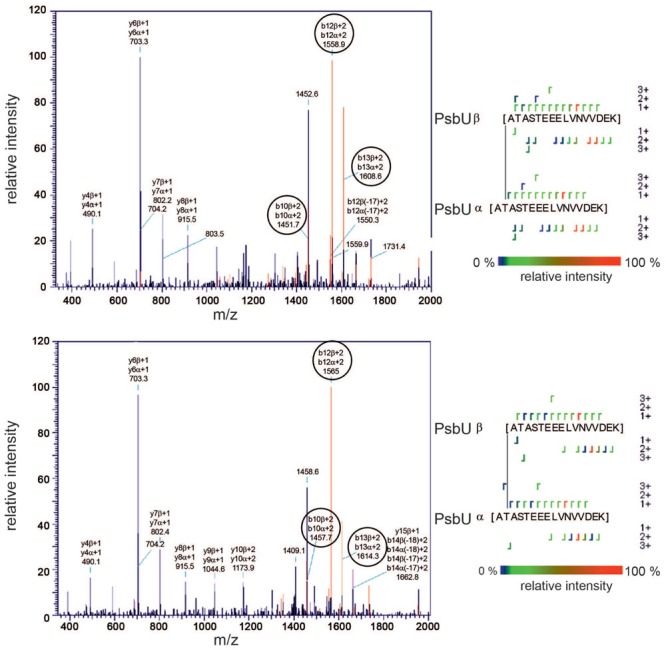
**Example of a correct assignment.** A cross-link between the N-termini of two PsbU subunits was identified unambiguously by two independent assignments for the light (H_12_-BS3, Score: 232; upper spectrum) and heavy (D_12_-BS3, Score: 238; lower spectrum) forms of the cross-linker. That the two fragmentation patterns are almost identical is obvious, but for some ions that contain the cross-linker (in this case only b-ions), the 12-Da mass shift between the light and heavy form is clearly discernible (black circles).

Hence, to avoid ambiguous assignments and to reduce the false discovery rate further, we included the deuterated cross-linker (D_12_-BS3) in our analysis. For each peptide containing a H_12_-BS3 modification the dataset should then include a second peptide with the same amino acid sequence modified by D_12_-BS3. The mass difference of 12 Da between the two versions of the cross-linker should be matched by the corresponding precursor ions and, due to the nearly identical chemical properties of H_12_-BS3 and D_12_-BS3, both peptides are expected to exhibit a very similar retention time and the same pattern of fragment ions. So, we considered only those peptides that fulfilled the following three criteria: (1) identification in the H_12_-BS3 and D_12_-BS3 modified form, (2) simultaneous elution, and (3) similarity of fragment-ion patterns. Using this more stringent approach, we identified 10 intermolecular and five intramolecular cross-links (Supplementary Table S1 and Supplementary Figures S1–S19). In addition to the similar fragment *patterns* of the peptides cross-linked with H_12_-BS3 and D_12_-BS3, the fragment *masses* in both spectra provide additional information which strengthens the validity of the assignment: While ions that do not include the cross-linking BS3 molecule exhibit identical masses in both spectra (e.g., y6α/β + 1), fragments containing the cross-linker show the predicted mass shift of 12 Da/z (e.g., b10α/β + 2, b12α/β + 2, and b13α/β + 2) as illustrated for the PsbU–PsbU cross-link (**Figure 4**).

Finally, it is noteworthy that the number of identified peptides exceeds the number of cross-links because, as a result of missed cleavages and methionine oxidation, some of the peptides included the same cross-link (e.g., a cross-link was identified in the oxidized and the reduced form of amethionine-containing peptide). Moreover, highly abundant peptides showed broad elution peaks and were identified several times.

### Flexible Termini are the Preferred Cross-Linking Sites

**FIGURE 5:**
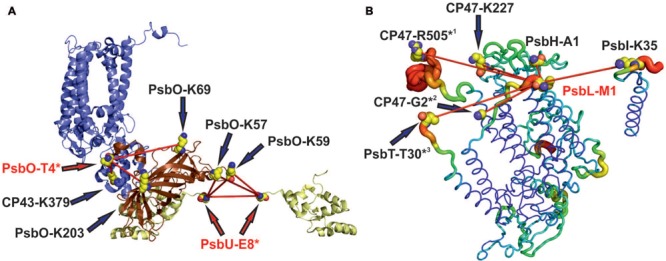
**Examples of cross-linking hotspots on the surface of PSII.** Of 15 experimentally derived cross-links, 14 involve protein N-termini, which are not resolved in the PSII crystal structure (PDB: 3ARC) due to their enhanced flexibility. **(A)** The N-termini of PsbO and PsbU are cross-linked to different amino acids at the lumenal PSII surface. **(B)** The N-terminus of PsbL is an example for a cross-linking hotspot on the cytoplasmic PSII surface. It is involved in six cross-links between PsbL and the adjacent subunits. Protein chains are labeled according to their B-factor with PyMOL (DeLano, 2002) from red (highest flexibility) to blue (rigid), and amino acids that are involved in a cross-link but are not resolved in the crystal structure are marked by an asterisk.

The high-resolution crystal structure of mature PSII should facilitate an *in silico* approach to the prediction of potential cross-links in the protein complex. We used the Xwalk algorithm (Kahraman et al., 2011) to calculate solvent-accessible surface distances between lysine residues in PSII. Potential cross-links were limited to a maximum distance of 35 Å, as the distance constraint for BS3 is 11.4 Å and the size of two lysine side-chains is 13 Å. The additional 10 Å was added to allow for conformational dynamics. Interestingly, Xwalk did not predict the majority of cross-links that were identified by the MS approach. The reason for this unexpected result becomes obvious on closer inspection of the crystal structure of PSII (Umena et al., 2011). Although the structure was solved with a high resolution of 1.9 Å, many of the protein termini are not resolved at all, due to their enhanced flexibility. On the other hand, in 14 of 15 cross-links a protein N-terminus provided at least one primary amine. This leads to the model of easy accessible and flexible cross-linking hotspots, like the N-termini of PsbO and PsbL (**Figure 5**), that readily react with the cross-linker. As a consequence, the partly fixed cross-linker scans the surface within a 35-Å radius for a second reactive side-chain (preferably lysine, but also serine or threonine). Thus, we never observed cross-links between protein–protein interfaces that are hidden in the complex. Instead, surface-exposed, flexible parts of PSII are the preferred cross-linking sites.

### Psb27 is Cross-Linked to the Center of the Lumenal CP43 Domain

**FIGURE 6:**
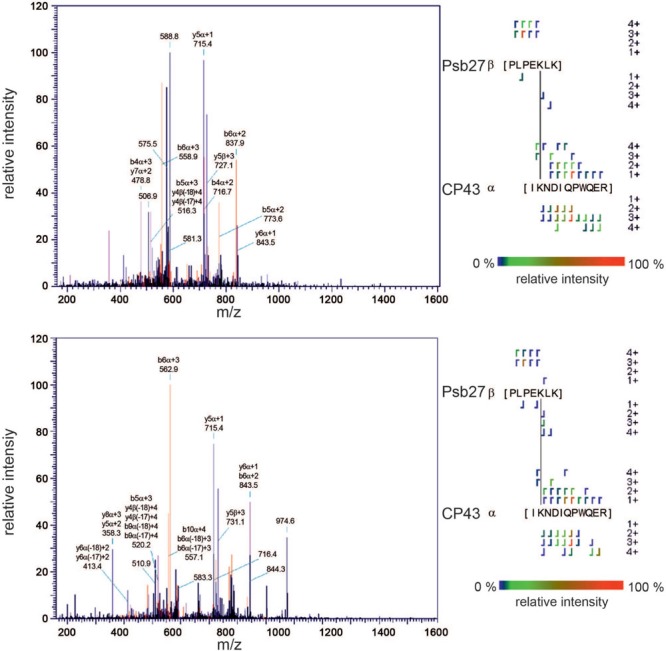
**Identification of a cross-link between Psb27 and CP43.** An intermediate Psb27-PSII complex (Nowaczyk et al., 2006) was labeled with isotopically coded BS3 and analyzed by MS. The MS2 fragmentation patterns for a peptide that includes a cross-link between Psb27-K91 and CP43-K381 formed by the light (upper spectrum, Score: 234) and the heavy (lower spectrum, Score: 178) form of the cross linker are shown. Masses of the precursor ions: 2388.329 and 2400.404 (*z* = 4); Examples of identical ions: y5α, y6α; Examples of ions that contain the cross-linker: y5β, b6α.

To probe the interaction of Psb27 with PSII, the corresponding monomeric intermediate complex without the extrinsic proteins PsbO, PsbU, and PsbV (Nowaczyk et al., 2006) was treated with the isotopically coded cross-linker and analyzed by mass spectrometry. Only one cross-link involving Psb27 was found, based on the three criteria for a reliable cross-link defined previously by our validation approach. The results shown in **Figure 6** clearly demonstrate the presence of a cross-link between Psb27-K91 and CP43-K381. The precursor ions of the light and heavy peptides (2388.329/2400.404 Da, *z* = 4) elute at the same time, and show a mass difference of 12 Da. Fragmentation of both precursor ions leads to high-quality MS2 spectra and reliable assignment of the fragmentation pattern (Score: 234/178). Some of the fragment ions exhibit identical masses (e.g., y5α/y6α), whereas others differ in m/z due to the presence of the cross-linker (e.g., y5β, b6α). In general, the intensity of assigned fragment ions is comparable between the two spectra, while unassigned fragments (e.g., 974.6) are mainly present in only one of the two spectra.

CP43-K381 is localized at the tip of the large lumenal domain of CP43, which hampers the identification of the exact Psb27 binding position. Being the only structural constraint within a radius of 35 Å, the cross-link allows for localization of Psb27 at the PsbO binding site, but also on the opposite side of the CP43 domain at the PsbV binding position. Interestingly, two other cross-links involving CP43-K381 (CP43-K323, CP43-K339) that are not present in the dataset for the mature PSII complex were identified in the Psb27-PSII dataset (Supplementary Table S2, Supplementary Figures S20–S29). This may indicate that CP43-K381 forms a cross-linking hotspot unless positions K323/K339 are shielded by PsbO/PsbU, and the whole domain might also be more flexible without the extrinsic proteins. Nevertheless, more structural constraints are necessary for the secure localization of Psb27 in order to distinguish at least between its binding to the PsbO or PsbV binding site onthe PSII surface.

### Psb27 Interacts with the Lumenal Domains of CP43, D1, D2, and CP47

**FIGURE 7:**
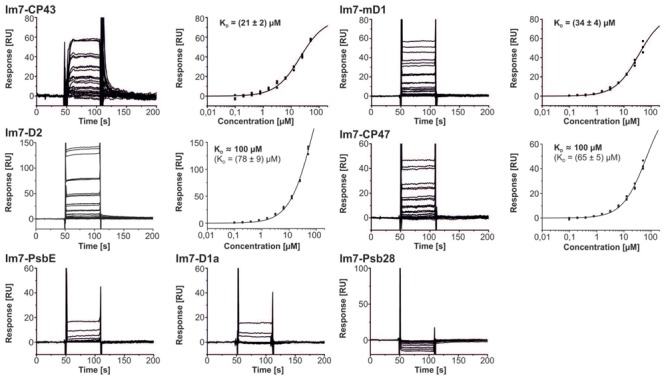
**SPR interaction analysis of Psb27.** Recombinant Psb27 was probed for binding to various lumenal PSII domains (CP43, CP47, D2, mD1, D1a, and PsbE) and cytoplasmic Psb28 as negative control. In the case of CP43, mD1, D2, and CP47, triplicate sensograms for each analyte concentration reveal reproducible, concentration-dependent steady-state binding responses that can be fitted to a one-site binding isotherm (*K*_D_ given with SEs). The highest affinities were measured for CP43 (21 ± 2 μM) and mD1 (34 ± μM), whereas lower affinities were determined for CP47 and D2 (∼100 μM). Analysis of D1a, PsbE, and the Psb28 control detected only weak binding at the highest concentrations.

For further analysis of the interaction between PSII and Psb27, recombinant Psb27 was probed for binding to various lumenal PSII domains (CP43, CP47, D2, mD1, D1a, PsbE) and a cytoplasmic control (Psb28) in an *in vitro* approach that is based on SPR spectroscopy (Cormann et al., 2014). The screening resulted in reproducible, concentration-dependent binding responses for CP43, CP47, D2, and the mature D1 C-terminus (mD1), each of which was fitted to a one-site binding isotherm (**Figure 7**). The highest affinities were measured between Psb27 and CP43 (21 ± 2 μM) and Psb27 and mD1 (34 ± 4 μM), whereas binding to CP47 and D2 occurred with considerably lower affinities (*K*_D_ ≈ 100 μM). No binding or very low binding responses were observed for PsbE, the D1 a-loop (D1a), or Psb28. These results support a localization of Psb27 at the PsbV-binding position, mainly driven by contacts to CP43 and D1, rather than by binding to the D2/D1a site. Finally, the ClusPro server 2.0 was used to predict the binding position of Psb27 on the PSII surface in an *ab initio* modeling approach and to compare the results with the experimentally derived constraints. Models were calculated based on the crystal structures of Psb27 (PDB code: 2Y6X) and PSII (PDB code: 3ARC) after removal of the extrinsic proteins, but none of the deduced models explains the simultaneous contacts to D1, D2, CP43, and CP47 (data not shown) detected by SPR spectroscopy.

## Discussion

The combination of chemical cross-linking with mass spectrometry is a very powerful tool for the structural analysis of intact protein complexes, but it is also challenging to use (Seebacher et al., 2006; Sinz, 2006; Petrotchenko and Borchers, 2010; Stengel et al., 2012; Bricker et al., 2015). Careful analysis of the results is necessary to avoid misinterpretation of the data, as the number of possible assignments for cross-linked peptides increases exponentially with the number of peptides in the database, and the complexity of the sample. If multiple post-translational modifications are additionally allowed for in the database search, the proportion of false positives will increase dramatically (Chen et al., 2009; Baker et al., 2010).

Therefore, to reduce the complexity of the samples as much as possible, we chose to work with highly purified PSII particles and restricted the data analysis to only one common post-translational modification (methionine oxidation) and one cross-link per peptide, and we employed an isotopically coded cross-linker that permits further validation of the assignments (Seebacher et al., 2006; Petrotchenko and Borchers, 2010). This required the use of two runs of StavroX for each dataset to independently identify cross-linked peptides with light and heavy forms of the cross-linker, which were added in a 1:1 ratio to each sample analyzed. Only peptides that were identified with high confidence in both runs, eluted at the same time in each case, and exhibited comparable fragmentation patterns, were considered as positive identifications. This workflow was further validated by correlation of the results with the known crystal structure of the mature PSII complex. Cross-links that matched our criteria were mapped to the high-resolution crystal structure of PSII, and inspected for plausible reaction partners within range of the cross-linker (<35 Å) and for steric hindrance. Moreover, all identified cross-links were examined for cleavage at the cross-link site, as trypsin, the most commonly used protease in proteomic studies, is unable to cleave C-terminal to modified lysine residues (Hitchcock et al., 2003; Prudova et al., 2010). All identified cross-links appeared feasible and consistent with the PSII structure.

Interestingly, almost all identified cross-links included at least one natural N-terminus as a cross-linking site, which is not unreasonable, as termini are usually the most flexible parts of proteins. This enhanced flexibility may increase the probability that the cross-linker will attach and, once fixed, can scan the surface within the distance accessible to it for a reaction partner, which might be located in a less flexible part of the protein. The preference of the BS3 cross-linker for flexible regions of proteins has previously been observed in a cross-linking and mass spectrometry study of chloroplast ATP synthase (Schmidt et al., 2013). Interestingly, MassMatrix, another commonly used software package for the analysis of cross-linked peptides (Xu and Freitas, 2009), does not consider cross-linked protein termini (Sriswasdi et al., 2014).

The validated workflow was then used for analysis of the intermediate Psb27-PSII complex. One cross-link between Psb27-K91 and CP43-K381 was identified with high confidence, and restricts the binding site of Psb27 to the vicinity of the lumenal CP43 domain. This result also points to one limitation of the cross-linking approach. Although the cross-link was identified with high confidence, its impact on the probabilistic determination of the binding site is relatively low. One structural constraint is not sufficient to fix the binding site, but even with more cross-links, it might still be difficult – depending on their positions – to localize the protein, since the long spacer length of BS3 introduces an inherent fuzziness. From a structural point of view, a zero-length cross-linker like EDC would be more appropriate, but interpretation of zero-length cross-linking MS data and application of differential isotopic labeling is also a much more complex undertaking (Rivera-Santiago et al., 2015).

Therefore, we turned to SPR-based mapping for more precise identification of the Psb27 binding site on PSII – an approach that has been used successfully to localize the PSII assembly factor CyanoP on the lumenal PSII surface (Cormann et al., 2014). This method relies on PSII peptides and domains that are expressed by *in vitro* translation, captured via the strong binding of the Im7 fusion partner to its cognate binding partner DNaseE7 and probed individually for interaction with the protein of interest (Cormann et al., 2014). Although, it is unknown if the PSII domains adopt native structures, this approach has been validated similarly using the high-resolution PSII crystal structure (Umena et al., 2011). In our previous study, we have shown that the extrinsic subunits PsbV and PsbO only bind to the lumenal domains which provide a broad interaction interface in the complex structure, whereas the other domains showed no or very weak binding responses (Cormann et al., 2014).

**FIGURE 8:**
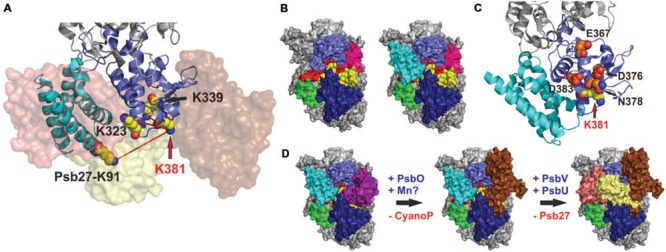
**Localization of Psb27 at the PsbV binding position on PSII. (A)** CP43-K381 becomes a cross-linking hotspot (CP43-K232, CP43-K339, and Psb27-K91) in the Psb27-PSII intermediate complex, whereas in the mature complex the whole CP43 domain (light blue) is masked and fixed by attachment of the extrinsic proteins PsbO (brown), PsbV (salmon), and PsbU (pale yellow). **(B)** Left: Lumenal PSII surface (CP43 domain: light blue; CP47 domain: dark blue; mD1: red; D1a: pink; D2: yellow; PsbE: green) without the extrinsic proteins. Right: The position of Psb27 (cyan) was modeled based on the cross-link between Psb27-K91 and CP43-K381, the results of the SPR analysis and the membrane attachment of its N-terminus. The position differs from that in a previous model, which localizes Psb27 more distally and partially overlapping the PsbO binding position (Liu et al., 2011a). **(C)** Amino-acid residues of CP43 (D383, E367, D376, and N378) that are in close proximity to CP43-K381 and are protected from chemical modification after binding of Psb27, according to the results of a footprinting approach (Liu et al., 2013). This model appears both incompatible with the previous model (Liu et al., 2011a) and inconsistent with the position of Psb27 in our model. **(D)** Proposed scheme for assembly of the extrinsic part of PSII. The interaction of Psb27 with the C-terminus of D1 in an intermediate PSII complex, and the simultaneous binding of CyanoP – which is speculative but possible (Cormann et al., 2014) – might imply a function for Psb27 during assembly and photoactivation of the manganese cluster. Subsequently, PsbO replaces CyanoP after incorporation of manganese and afterward Psb27 is replaced by PsbV and PsbU in the proposed scheme.

Here, we observed binding of Psb27 to lumenal domains of CP43 and CP47, as well as to the C-termini of D1 and D2, which is consistent with the CX-MS result. Moreover, binding to CP43 and D1 seems to be the main driving force for the interaction with the PSII surface, as the binding affinities for both are considerably higher than those for CP47 and D2. These results would favor a localization of Psb27 at the PsbV binding position on PSII (**Figures 8A,B**), which is consistent with biochemical data. It has been shown previously that highly purified Psb27-PSII complexes from *T. elongatus* are devoid of PsbO, PsbV, and PsbU, and that *in vitro* reconstitution of the Psb27-PSII complex with the isolated extrinsic proteins is apparently impossible under the conditions tested (Nowaczyk et al., 2006). However, purified PSII complexes isolated from *Synechocystis* sp. PCC6803 via His-tagging of Psb27 have been found to contain considerable amounts of PsbO, but not PsbV or PsbU (Liu et al., 2011b). These data suggest that simultaneous binding of PsbO and Psb27 is possible, albeit with less stable association of PsbO relative to the mature complex, but simultaneous binding of Psb27 and PsbV or PsbU seems rather unlikely.

Moreover, we have demonstrated a direct interaction of Psb27 with mD1, which argues against the more distal binding position previously proposed for Psb27 on the basis of cross-linking data (Liu et al., 2011a), even if the whole CP43 domain were more flexible in the intermediate PSII complex lacking the adjacent extrinsic proteins. Interestingly, we observed that the CP43-K381 residue involved in the cross-link with Psb27 acts as a cross-linking hotspot on the lumenal surface of the PSII intermediate, but not in the mature complex (**Figure 8A**). The accessibility of CP43-K381/K323/K339 in the Psb27-PSII complex is also inconsistent with the localization of Psb27 based on a chemical footprinting approach (Liu et al., 2013). These authors reported that several residues in the CP43 loop domain (Glu367, Asp376, Asp378, and Asp383) became inaccessible to the solvent upon binding of Psb27, whereas they should be solvent-accessible according to our model (**Figure 8C**). We cannot explain the specific factors that have led to three different experimentally derived models for binding of Psb27 to PSII, but the problems may be related to the experimental conditions used, as mentioned earlier (Mabbitt et al., 2014) or to the interpretation of MS data. Strikingly, one of the cross-linked peptides (Psb27K63↔CP43D321) identified by Liu et al. (2011a) is apparently cleaved by trypsin C-terminal to Psb27K63, although it should be protected by the cross-link. Furthermore, both presented cross-links (Psb27K58↔CP43D215 and Psb27K63↔CP43D321) are supposed to contain post-translational modifications (triple methylation, deamidation, formation of methyl esters) that have not otherwise been reported for cyanobacterial samples to the best of our knowledge, and which cannot be explained as by-products of sample preparation (Liu et al., 2011a). One reason for these contradictions could be a false positive identification by MassMatrix (Xu et al., 2008; Xu and Freitas, 2009), the software which was used in the 2011 study. The probability of false positive identifications is given by the pp-value, which is calculated by the software for each identification (Xu et al., 2008; Xu and Freitas, 2009). Liu et al. (2011a) used a minimal pp-score of 5 for the cross-link analysis, which might be compatible with a high proportion of false positive identifications (Xu et al., 2008; Xu and Freitas, 2009).

However, localization of Psb27 at the PsbV binding position is in agreement with concurrent binding of CyanoP, which is localized at the PsbO binding site (Cormann et al., 2014). Both proteins are able to bind simultaneously to an intermediate PSII complex that plays a role in PSII biogenesis/repair and both interact with the C-terminus of D1 (Cormann et al., 2014), which suggest a cooperative role in manganese cluster assembly (**Figure 8D**). It has been shown previously that Psb27 facilitates assembly of the manganese cluster (Roose and Pakrasi, 2008) and plays a role in the structural integrity of the manganese binding site (Grasse et al., 2011), but the model and the sequence of events (**Figure 8D**) are speculative, as it is not clear whether the complexes depicted are actually formed *in vivo*. Binding of Psb27 to PSII is further constrained by its N-terminal lipid modification (Nowaczyk et al., 2006), which is difficult to consider by an *in vitro* approach. Integration of additional *in vivo* data and in particular crystallization of – in case of Psb27 – relatively stable intermediate complexes would further reveal the structural and functional relationships between Psb27 and PSII during biogenesis and repair.

## Conclusion

We have shown that *in silic*o molecular docking does not seem to be suitable for the localization of auxiliary proteins on the lumenal PSII surface, at least with the tested software and where no further experimental data are available. False positive results are also an important issue in the identification of cross-links by mass spectrometry. Therefore, we have developed a CX-MS approach for the structural analysis of intermediate PSII complexes that is based on high-resolution mass spectrometry and isotope-coded cross-linkers. Validation of the method by comparison with the PSII crystal structure identified only cross-links that are consistent with the structure, and analysis of an intermediate Psb27-PSII complex corroborated binding of Psb27 adjacent to the lumenal CP43 domain and the C-terminus of D1. In particular the combination of the two complementary methods – CX-MS and SPR based mapping – facilitates a detailed structural characterization of unstable intermediate complexes that are part of the intricate life cycle of PSII.

## Materials and Methods

### PCR and Molecular Cloning

The coding sequence of the *psb27* gene (*tll2462*) of *T. elongatus* (without the segment encoding the signal peptide and the N-terminal cysteine) was amplified by PCR, using the primer oligonucleotides: GAAGATGGTCTCTGCGCCGCCAATGTGCCTACGG (forward) and GAATAAGGTCTCCTATCAGGACTTCGCTTCGCG (reverse). After digestion with *Eco31*I, the product was inserted into the pASK-IBA35plus vector (IBA).

To generate an expression vector for Psb28 fused to the ColE7 immunity protein (Im7), the *psb28* gene of *T. elongatus* (*tlr0493*) was amplified by PCR using the primers GGAATTCCATATGGGTGCAATGGCAGAAATTC (forward) and CGAATTCCCCGAGAGTTCTCAGACTTCTG (reverse). Subsequently, the gene sequence was inserted into the plasmid pIVEX24IN (Cormann et al., 2014) via the *Nde*I and *Sma*I restriction sites. Construction of expression templates for Im7 fusions to the lumenal PSII domains is described elsewhere (Cormann et al., 2014).

### Cell-Free Protein Expression

For cell-free expression of Im7-tagged domains of CP43 (residues 293 – 424), CP47 (271 – 446) D1 (D1a: 54 – 113; mD1: 295 – 344), D2 (294 – 352), and PsbE (48 – 84), we used the RTS100 system (5Prime) as described by Cormann et al. (2014).

### Heterologous Protein Overexpression and Purification

Heterologous overexpression of Psb27 was performed in *Escherichia coli* Overexpress C43 cells (Lucigen). After transformation of C43 cells with the Psb27 plasmid, an overnight starter culture (50 ml LB medium supplemented with 1 % (w/v) glucose and 100 μg/ml ampicillin) was inoculated with a single colony. The culture was grown in vigorously shaken (200 rpm) flasks at 37°C. Then two flasks with 500 ml were each inoculated with a 5-ml aliquot of the overnight preculture and incubated at 200 rpm and 37°C. Psb27 expression was induced by the addition of anhydrotetracycline (200 μg/l) when the cultures had reached an OD_600_ of 0.6. After additional 16 h of incubation, the cells were harvested (4000 rpm, 20 min, 4°C, GSA rotor), resuspended in IMAC equilibration buffer (20 mM MES, 500 mM NaCl, 20 mM imidazole; pH 6.5) and disrupted by sonification. For purification of Psb27, the ÄKTA system (GE, Healthcare) was used. In the first step, the supernatant was applied to an equilibrated IMAC column (His Trap crude FF 5 ml, GE Healthcare) and washed with ten column volumes (CV) of IMAC equilibrating buffer. For elution, a linear gradient from 0 to 66% of IMAC elution buffer (20 mM MES, 500 mM NaCl, 500 mM imidazole; pH 6.5) was applied at a flow rate of 2 ml/min over four CV, and this was followed by five CV of 100% IMAC elution buffer. Fractions containing Psb27 were pooled and dialyzed twice against 1 l of IEC equilibration buffer (20 mM MES, pH 6.5). For the second purification step a ResourceS column (*CV* = 1 ml, GE Healthcare) was equilibrated with five CV of IEC equilibration buffer at a flow rate of 4 ml/min to reduce the conductivity to ≤4.5 mS/cm. A sample volume equivalent to the yield of protein from 1 l of expression culture was applied to the column, which was then washed with five CV of IEC equilibration buffer. For elution a linear gradient from 0 to 50% of IEC elution buffer (20 mM MES, 1 M NaCl; pH 6.5) was applied over 20 CV, followed by 100% elution buffer over 10 CV. The purified protein was dialyzed overnight against 1 l of MBS (20 mM MES, 150 mM NaCl; pH 6.5) and stored at –80°C.

The expression and purification of Psb28 fused to Im7 was performed according to Cormann et al. (2014).

### Isolation of PSII from *T. elongatus*

Isolation of PSII complexes from wild-type *T. elongatus* was carried out according to Kuhl et al. (2000).

### 4.5 Cross-Linking in Combination with Mass Spectrometry (CX-MS)

Isolated PSII complexes (2.4 μM) were incubated with a 1:1 mixture of BS3-H12/D12 (Creative Molecules Inc.) in buffer (20 mM MES/NaOH, 10 mM CaCl_2_, 0.03% (w/v) dodecyl-β-D-maltoside; pH 6.5) containing the cross-linker at a final concentration of 5 mM. After a 30-min incubation on ice, NH_4_HCO_3_ (100 mM) was added, and the reaction mixture was incubated for 15 min at room temperature to terminate the cross-linking reaction. Tryptic digestion and sample preparation were performed after protein precipitation by addition of cold acetone (Nowaczyk et al., 2011). Tryptic peptides were desalted with ZipTips (Millipore) and resuspended in buffer A (0.1% (v/v) formic acid in water). Samples were applied to an UPLC Symmetry C_18_ trapping column (5 μm, 180 μm × 20 mm) and subsequently transferred to an UPLC BEH C_18_ column (1.7 μm, 75 μm × 150 mm). Both columns (Waters, Milford, MA, USA) were driven by the nanoACQUITY gradient UPLC pump system (Waters) coupled to an Orbitrap Elite Velos Pro Hybrid FTMS mass spectrometer (Thermo Fisher Scientific Inc., Waltham, MA, USA) via a PicoTip Emitter (SilicaTip, 30 μm, New Objective, Woburn, MA, USA). The column oven was set to a temperature of 45°C and the spray voltage to 1.5–1.8 kV. Peptides were eluted with a multistep gradient of buffer A to buffer B (0.1% formic acid in acetonitrile) at a flow rate of 0.4 μl/min (0–5 min: 1% buffer B; 5–10 min: 5% buffer B; 10–175 min: 40% buffer B; 175–200 min: 99% buffer B; 200–210 min: 1% buffer B). The linear ion trap and orbitrap were operated in parallel, i.e., during a full MS scan on the orbitrap in the range of 300–2000 m/z at a resolution of 240,000. Tandem MS (MS/MS) spectra of the 20 most intense precursors were detected in the ion trap. The heated desolvation capillary was set to 200°C. The relative collision energy for collision-induced dissociation was set to 35%. Dynamic exclusion was enabled, with a repeat count of one and a 1-min exclusion duration window. Singly or doubly charged ions, as well as ions with an unassigned charge state, were rejected from MS/MS. StavroX v. 3.1.19 was used for MS/MS data interpretation (Götze et al., 2012) with the following settings: Protease cleavage sites: K, R; missed cleavages: K:3, R:1; Variable modifications: methionine oxidation (max. 2); cross-linker: BS3-H12/D12; cross-linked amino acids: 1. K, N-term.; 2. K, S, T, Y, N-term; precursor precision: 3.0 ppm; fragment ion precision: 0.8 Da; lower mass limit: 200 Da; upper mass limit: 5000 Da; S/N ratio: 2.0; ion types: b-and y-ions; neutral loss: only of identified fragments.

### Surface Plasmon Resonance Spectroscopy

Surface plasmon resonance measurements were performed with a Biacore 3000 instrument and CM5 sensor chips (GE Healthcare). Covalent binding of DNase E7 to the gold surface was done according to Hosse et al. (2009). The Gentle Elution Buffer (Thermo Scientific) was used for surface conditioning, with two consecutive 1-min injections and a flow rate of 60 μl/min. Details of the on-chip purification of the Im7 fusion proteins and the preparation of the reference surface are given in Cormann et al. (2014). For SPR interaction analysis with the lumenal PSII domains a dilution series from 50 μM to 100 nM of Psb27 in running buffer MBS-EP (20 mM MES, 150 mM NaCl, 3 mM EDTA, 0,05% (v/v) Surfactant P20; pH 6.5) was applied for 60 s to the reference and the active surface. Dissociation was monitored for 120 s, and afterward the surface was cleaned with a 1-minute injection of 1 M NaCl in MBS-EP. Each experiment was performed at a constant flow rate of 30 μl/min and at a temperature of 298 K.

### *Ab Initio* Docking

The Cluspro 2.0 Server (Comeau et al., 2004a,b) was used for *ab initio* docking calculations. It calculates various structures of the protein complex on the basis of protein structures in pdb files. In this case, monomeric PSII without the extrinsic subunits PsbU, PsbO, PsbV (PDB: 3ARC) (Umena et al., 2011) and the crystal structures of PsbV (PDB: 1MZ4) (Kerfeld et al., 2003) and Psb27 (PDB: 2Y6X) (Michoux et al., 2012) were used. The models of docked PsbV were then compared to the crystal structure.

### Cross-Link Prediction with Xwalk

The Xwalk algorithm (Kahraman et al., 2011) was used for the calculation of distances between solvent-accessible primary amines in the PSII crystal structure (Umena et al., 2011). These models were calculated without additional distance information.

## Author Contributions

KC designed and carried out most of the experiments, analyzed the data, and prepared the figures. MP was involved in surface plasmon resonance experiments and data analysis. MN conceived and supervised the project, analyzed the data, and wrote the manuscript, with contributions of all authors.

## Conflict of Interest Statement

The authors declare that the research was conducted in the absence of any commercial or financial relationships that could be construed as a potential conflict of interest.
