# Supplementary material for: Critical Assessment of Protein Cross-Linking and Molecular Docking: An Updated Model for the Interaction Between Photosystem II and Psb27
Source: Front Plant Sci. 2016 Feb 18;7:157. doi: 10.3389/fpls.2016.00157 (PMC4758025; doi:10.3389/fpls.2016.00157)
Supplement: Supplementary file 1 [file Data_Sheet_1.PDF]

Supplementary Material

Critical assessment of protein cross-linking and molecular docking: an updated model for the interaction between photosystem II and Psb27

Kai U. Cormann, Madeline Möller, Marc M. Nowaczyk*

*** Correspondence:** marc.m.nowaczyk@rub.de

1 Supplementary Tables

Table S1: Crosslinks identified in mature PSII

CL	Protein 1	Protein 2	AA1	AA2	Score(H12/D12)	Measured mass (H12/D12)	Dev H12/D12)
1	PsbH	PsbL	NT	NT	273/99	2077.062/2089.134	0.43/-1.09
2	PsbU	PsbU	NT	NT, T2, S4, or T5	232/238	3604.754/3616.830	1.73/1.50
3	PsbO	PsbU	K59	NT	226/238	2288.163/2300.233	1.50/-1.81
4	PsbO	PsbU	K57	NT	218/145	2615.293/2627.368	-0.23/-0.31
5	PsbO	CP43	NT	K379	191/103	1523.880/1535.953	0.33/-0.80
6	PsbO	PsbO	NT	K203	150/177	1711.971/1724.045	0.26/-0.31
7	PsbT	PsbL	K32	NT	100 /121	1499.790/1511.864	0.69/0.10
8	PsbO	PsbO	NT	K69	142/128	2029.143/2041.219	-0.78/-0.40
9	PsbV	PsbV	K30	NT	137/139	2827.516/2839.587	2.72/0.94
10	CP43	PsbI	K154	K35	82/105	1301.708/1313.782	1.89/0.38
11	CP47	PsbL	NT	NT	175/120	1785.874/1797.952	0.21/1.20
12	CP47	PsbL	K227	NT	98/129	1773.932/1786.006	0.01/-0.54
13	PsbI	PsbL	K35	NT	150/118	1654.820/1666.985	-0.91/-1.06
14	PsbV	PsbV	K129	NT	235/233	2964.594/2976.671	0.33/0.85
15	D2	CP47	NT	K227	66L /117	1530.935/1543.012	-1.19/0.27
Ambiguous crosslinks							
Ia	CP47	PsbL	K506	NT	175/111	1596.813/1608.891	-2.06/-0.15
Ib	PsbX	PsbL	S40	NT	166/82	1596.813/1608.891	-2.06/-0.15
IIa	CP47	PsbE	K506	NT	165/136	1430.761/1442.834	0.74/-0.40
IIb	PsbX	PsbE	S40	NT	159/105	1430.761/1442.834	0.74/-0.40

Protein: proteins involved in crosslink; AA: amino acids involved in crosslink; NT: N-terminus; Dev: deviation between calculated and measured mass (ppm)

Table S2: Crosslinks identified in the intermediate PSII-Psb27 complex.

NR	Protein1	Protein2	AA1	AA2	Score (H12/D12)	Measured mass (H12/D12)	Dev (H12/D12)
1	Psb27	CP43	K91	K381	234/178	2388.329/2400.404	0.09/-0.08
2	PsbI	CP43	K35	K457	252/256	2003.055/2015.130	-0.15/-0.41
3	CP43	CP43	K381	K323	338/204	3361.752/3373.835	0.60/2.92
4	CP43	CP43	K381	K339	227/194	3569.862/3581.933	2.43/1.29
5	PsbI	CP43	K33	K457	218/151	1856.998/1869.072	-0.08/-0.59
6	PsbT	CP47	K32	NT	151/57	1417.819/1429.896	-0.70/0.05
7	PsbH	PsbL	NT	NT	150/94	2077.060/2089.137	0.34/0.40
8	PsbT	PsbL	K32	NT	148/130	1499.788/1511.863	-0.35/-0.93
Ambiguous crosslinks							
Ia	CP47	PsbL	K506	NT	116/121	1596.812/1608.890	-2.63/-0.72
Ib	PsbX	PsbL	S40	NT	114/96	1596.812/1608.890	-2.63/-0.72

Protein: proteins involved in crosslink; AA: amino acids involved in crosslink; NT: N-terminus; Dev: deviation between calculated and measured mass (ppm)

2 Additional Supplementary Files:

The file ‘Psb27_model.pdb’ contains the modified coordinates for the position of Psb27 (original PDB code: 2Y6X) relative to PSII (PDB code: 3ARC). This is a hand-made model to illustrate a feasible position of Psb27 that is in accordance with the experimental data.

3 Supplementary Figures

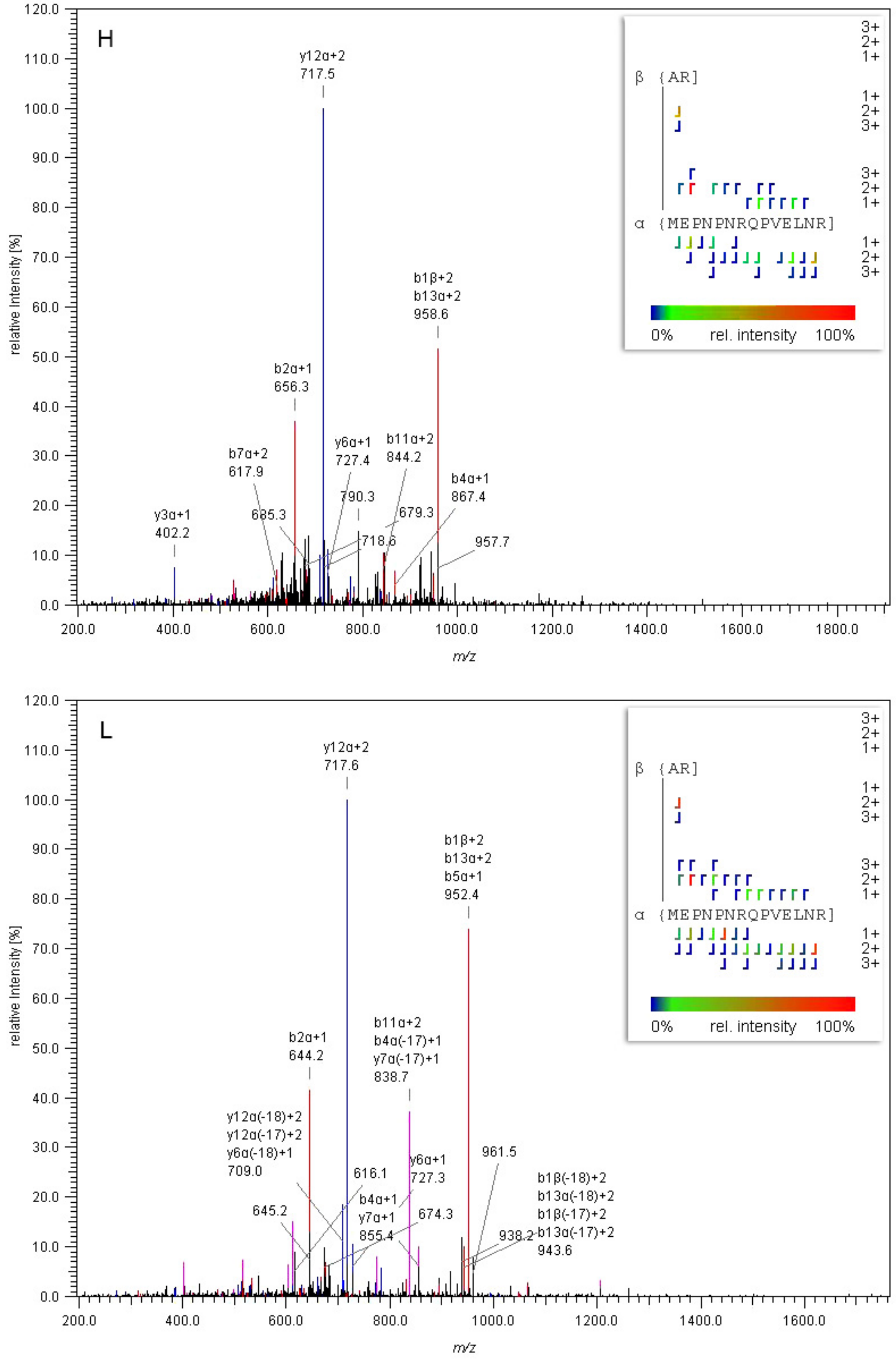

Supplementary Figure 1. Crosslink 1 of table S1. Identified b-ions (red), y-ions (blue) and unidentified signals (black) are indicated in the spectrum. Purple signals represent either b- or y-ions. H: heavy; L: light form of the cross linker.

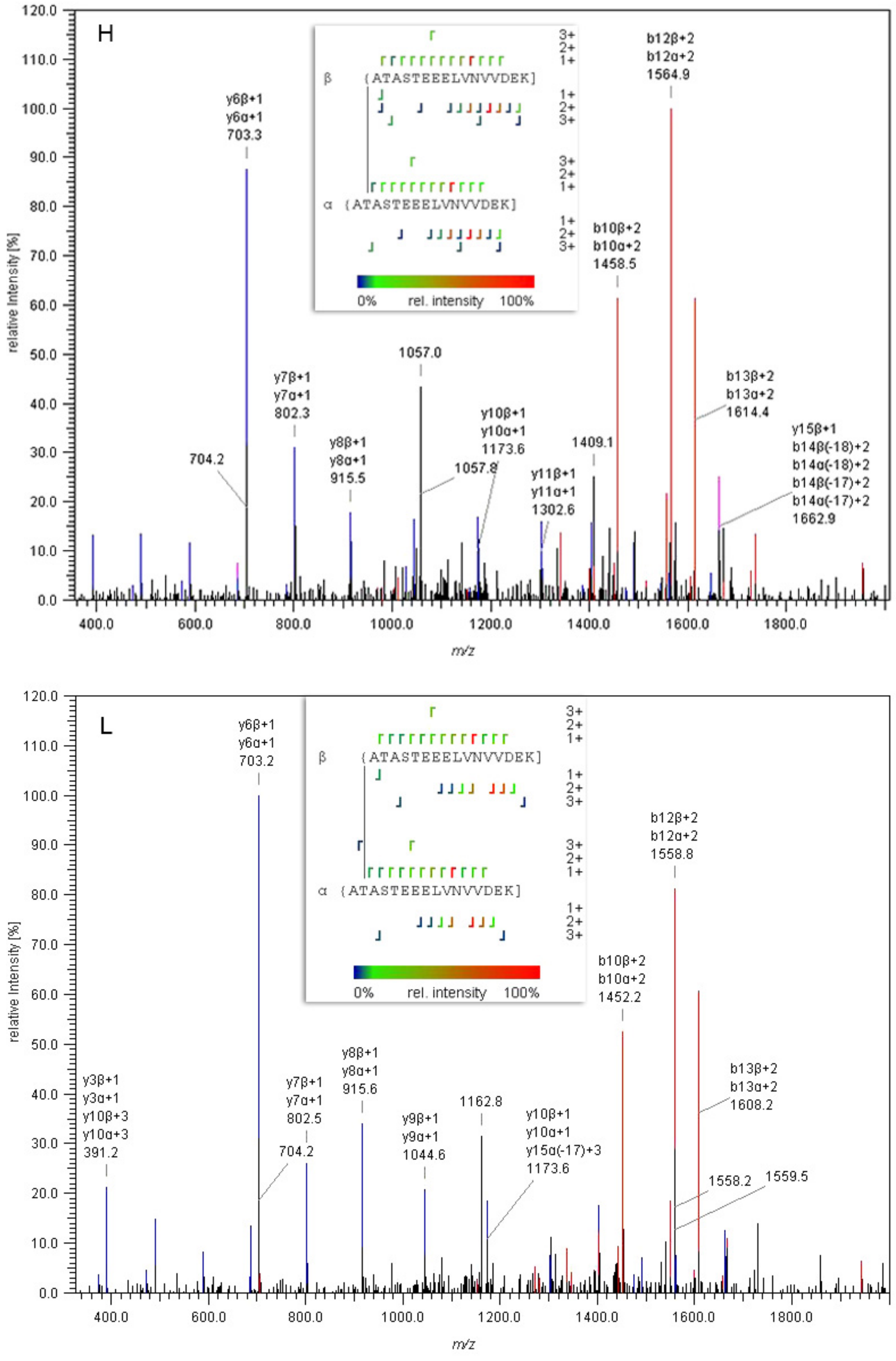

Supplementary Figure 2. Crosslink 2 of table S1. Identified b-ions (red), y-ions (blue) and unidentified signals (black) are indicated in the spectrum. Purple signals represent either b- or y-ions. H: heavy; L: light form of the cross linker.

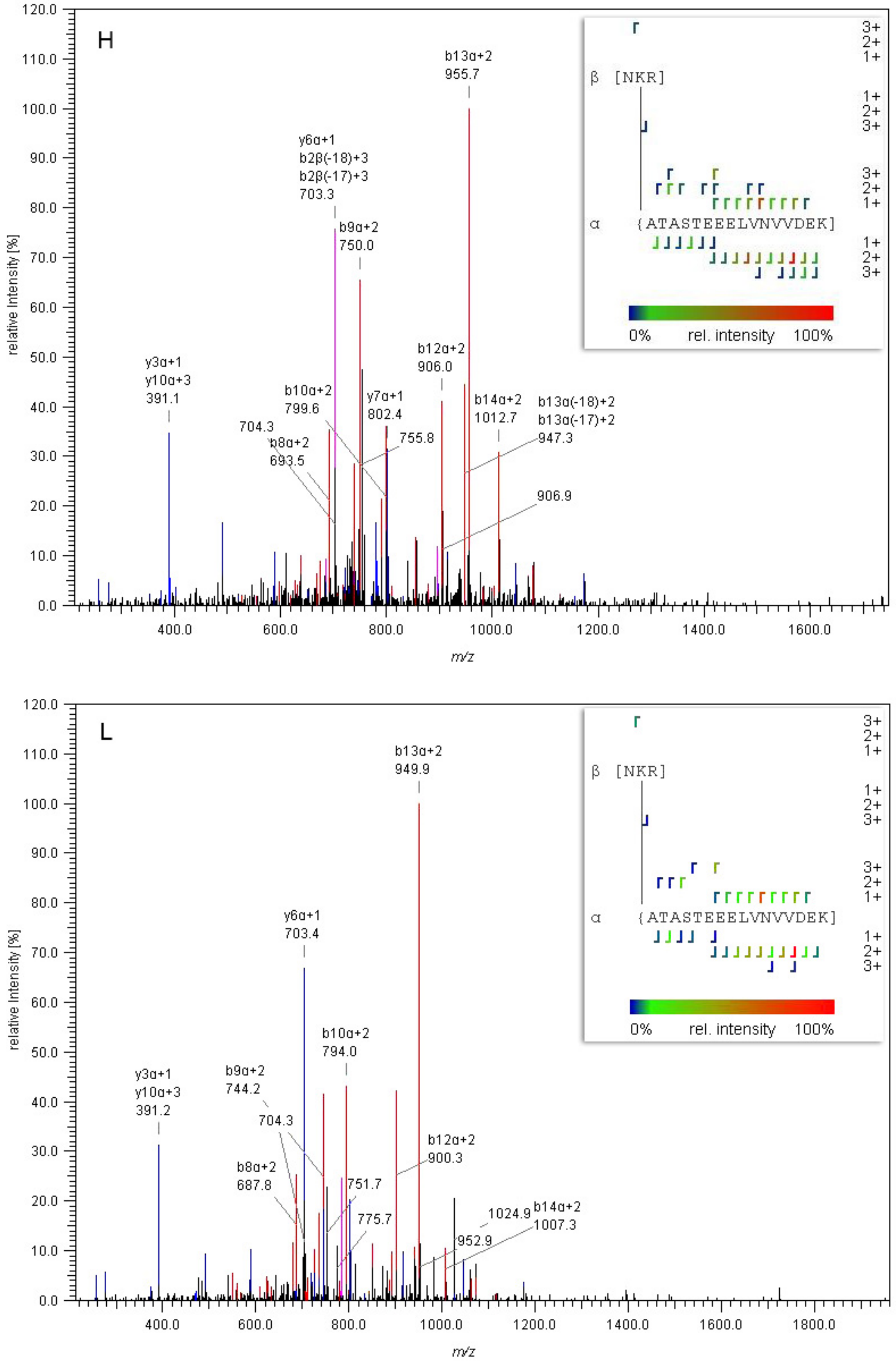

Supplementary Figure 3. Crosslink 3 of table S1. Identified b-ions (red), y-ions (blue) and unidentified signals (black) are indicated in the spectrum. Purple signals represent either b- or y-ions. H: heavy; L: light form of the cross linker.

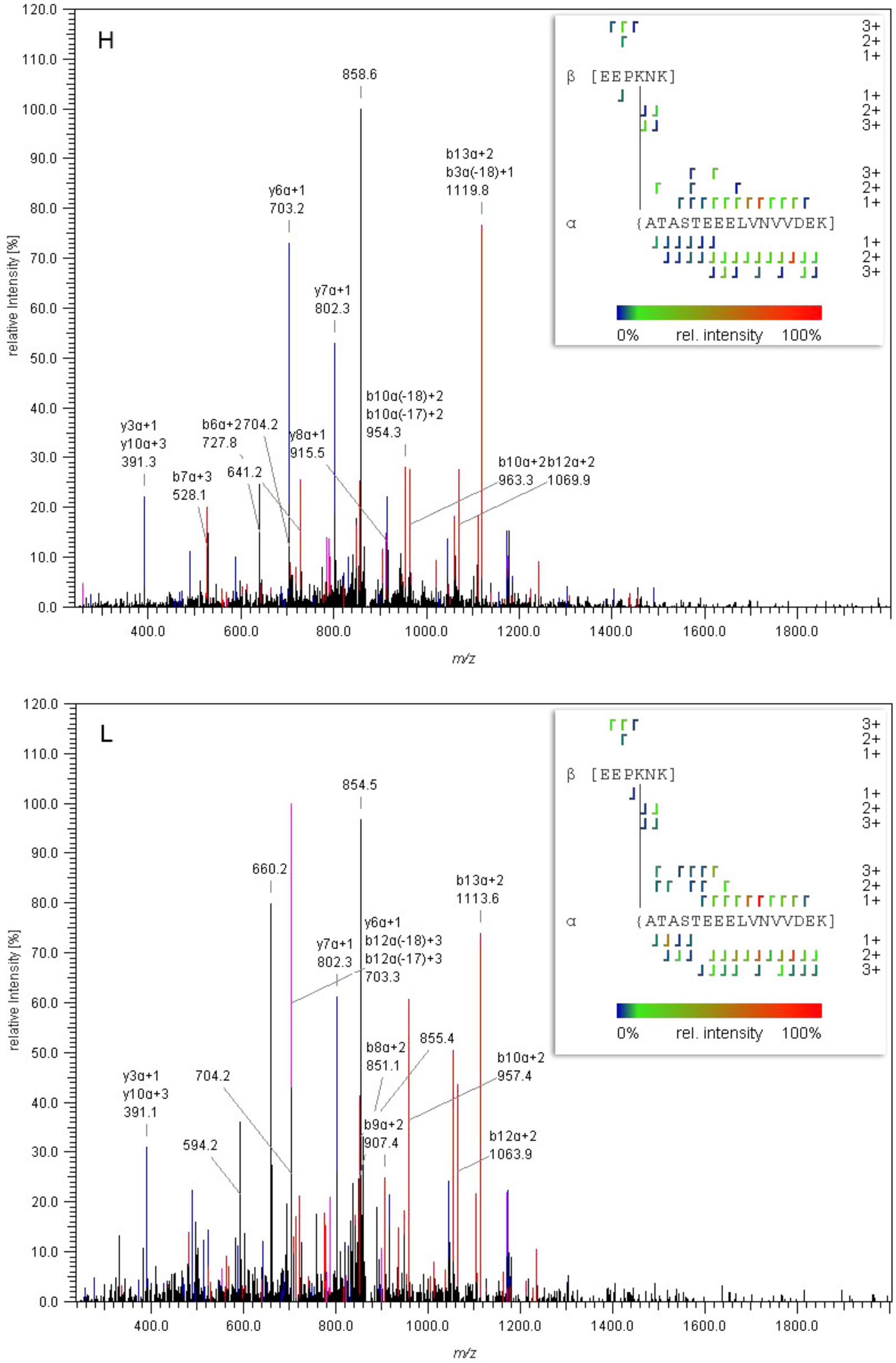

Supplementary Figure 4. Crosslink 4 of table S1. Identified b-ions (red), y-ions (blue) and unidentified signals (black) are indicated in the spectrum. Purple signals represent either b- or y-ions. H: heavy; L: light form of the cross linker

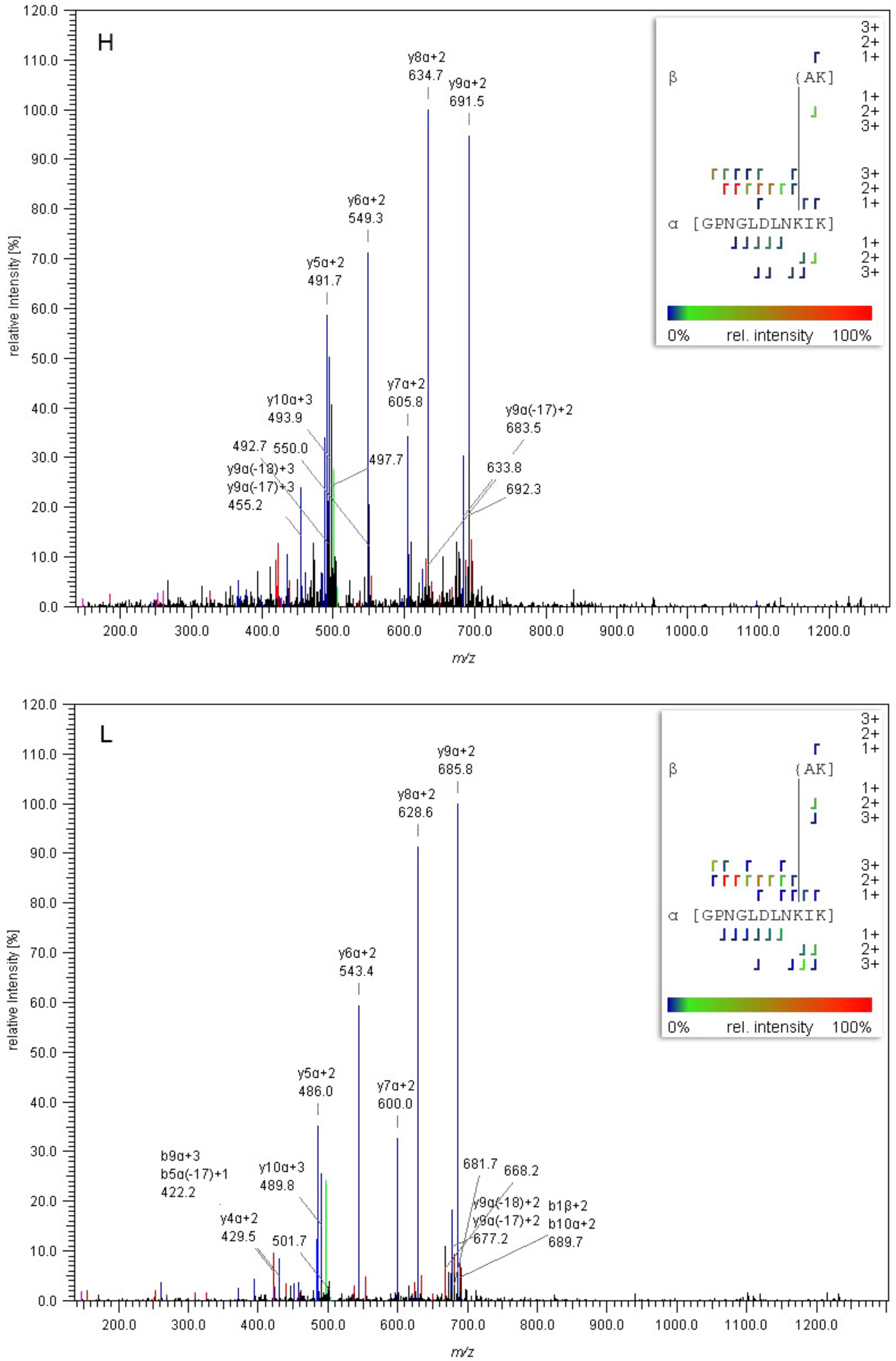

Supplementary Figure 5. Crosslink 5 of table S1. Identified b-ions (red), y-ions (blue) and unidentified signals (black) are indicated in the spectrum. Purple signals represent either b- or y-ions. H: heavy; L: light form of the cross linker

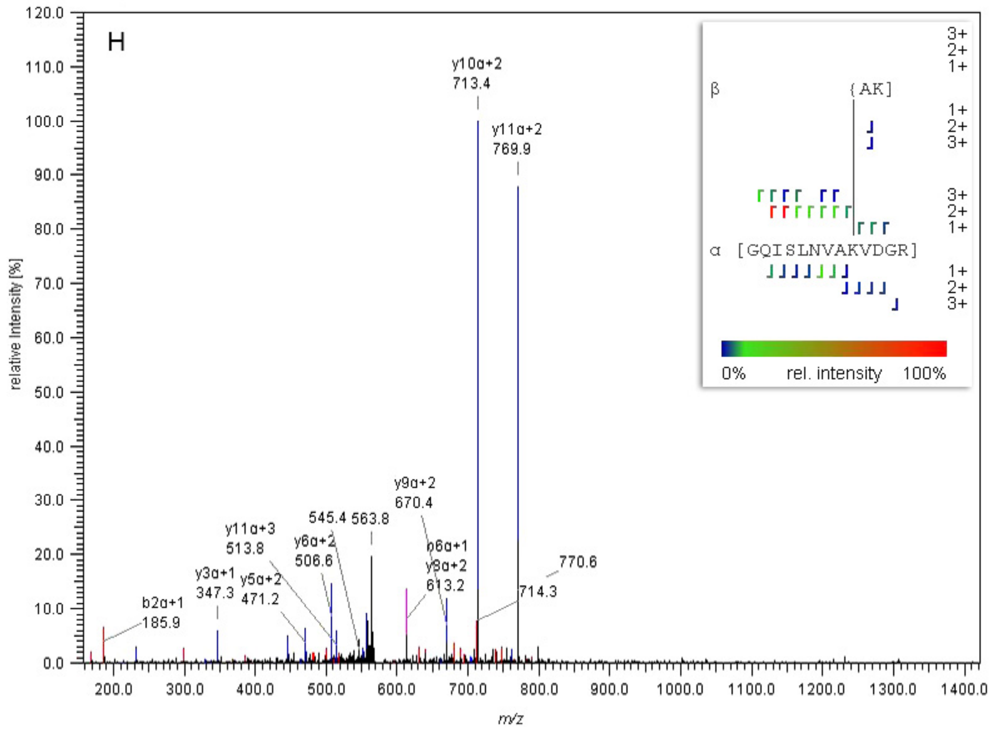

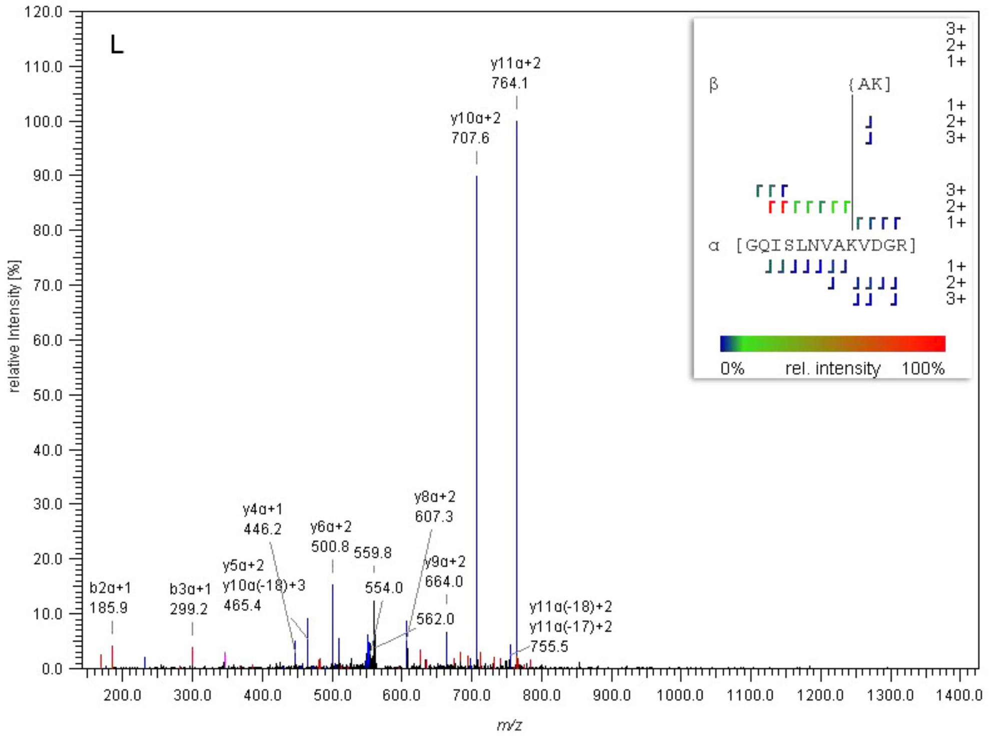

Supplementary Figure 6. Crosslink 6 of table S1. Identified b-ions (red), y-ions (blue) and unidentified signals (black) are indicated in the spectrum. Purple signals represent either b- or y-ions. H: heavy; L: light form of the cross linker

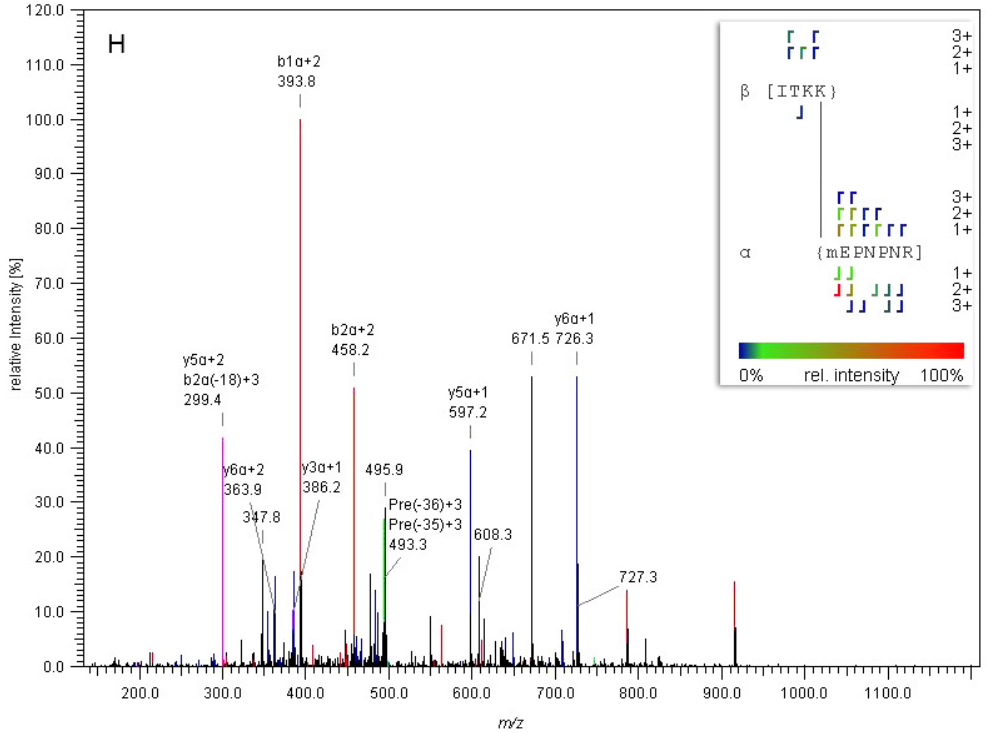

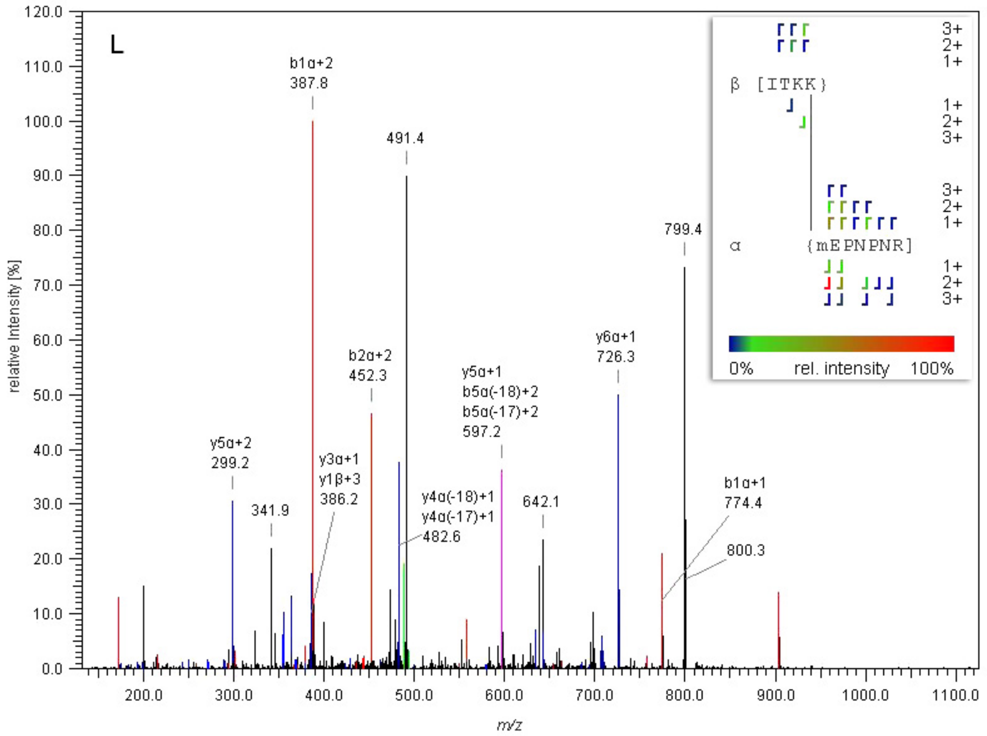

Supplementary Figure 7. Crosslink 7 of table S1. Identified b-ions (red), y-ions (blue) and unidentified signals (black) are indicated in the spectrum. Purple signals represent either b- or y-ions. H: heavy; L: light form of the cross linker

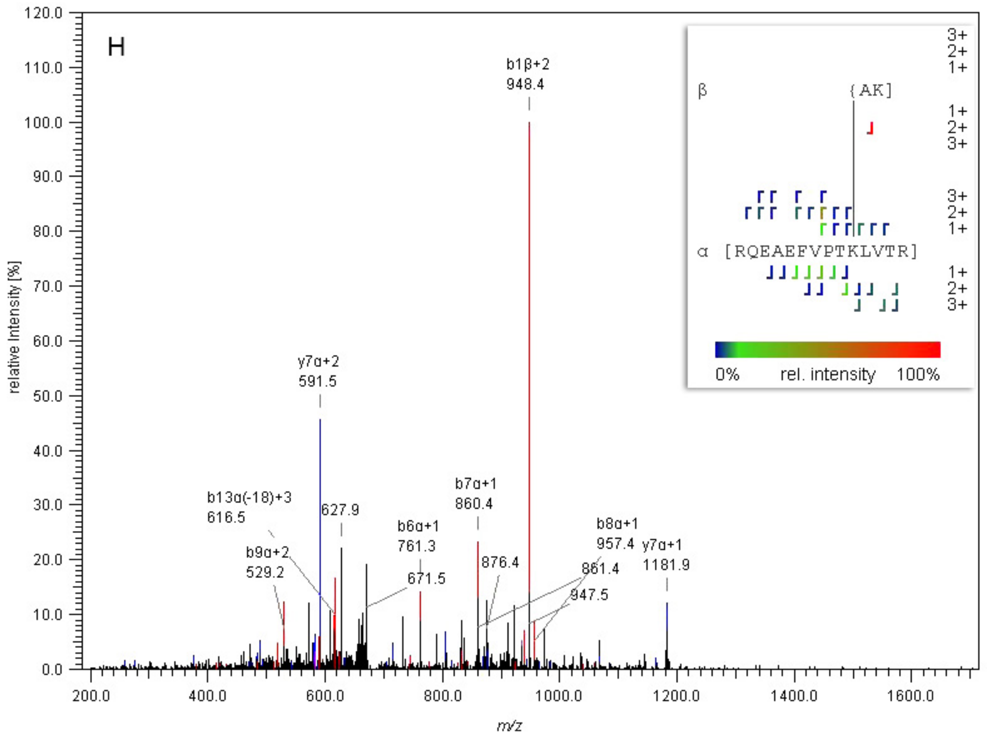

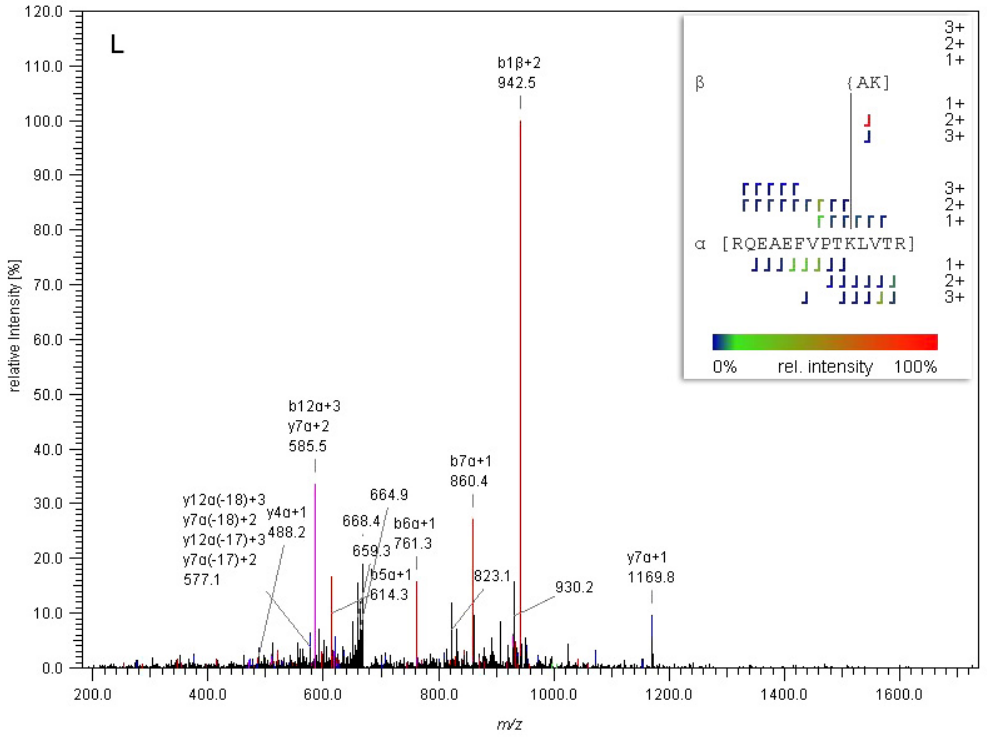

Supplementary Figure 8. Crosslink 8 of table S1. Identified b-ions (red), y-ions (blue) and unidentified signals (black) are indicated in the spectrum. Purple signals represent either b- or y-ions. H: heavy; L: light form of the cross linker

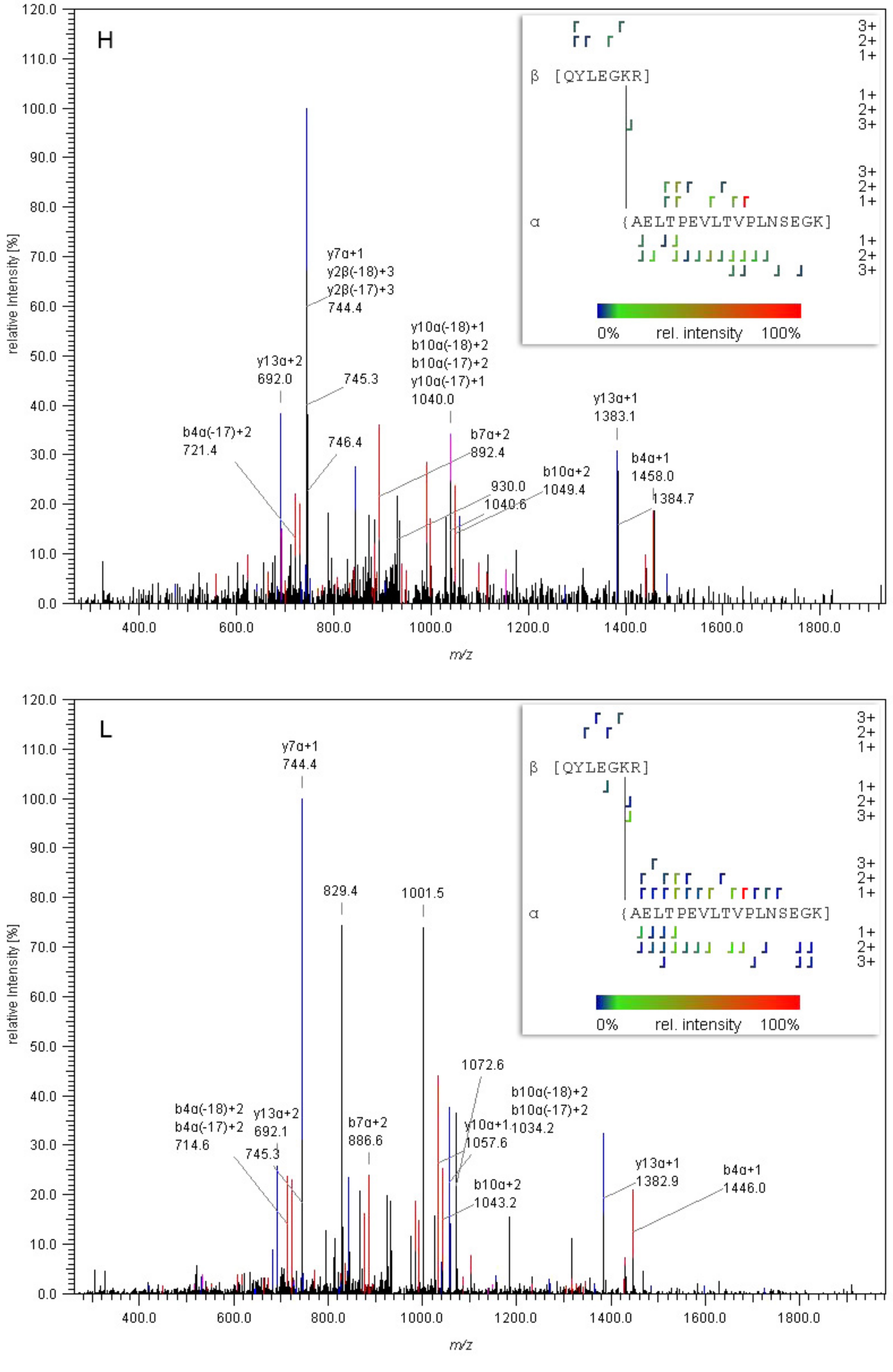

Supplementary Figure 9. Crosslink 9 of table S1. Identified b-ions (red), y-ions (blue) and unidentified signals (black) are indicated in the spectrum. Purple signals represent either b- or y-ions. H: heavy; L: light form of the cross linker

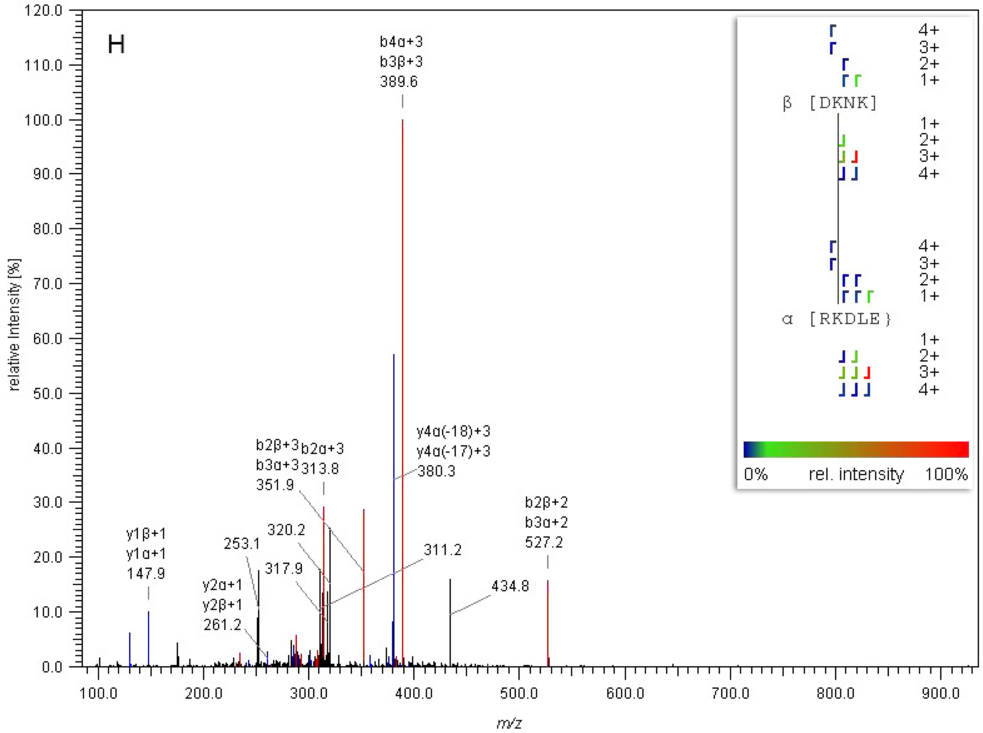

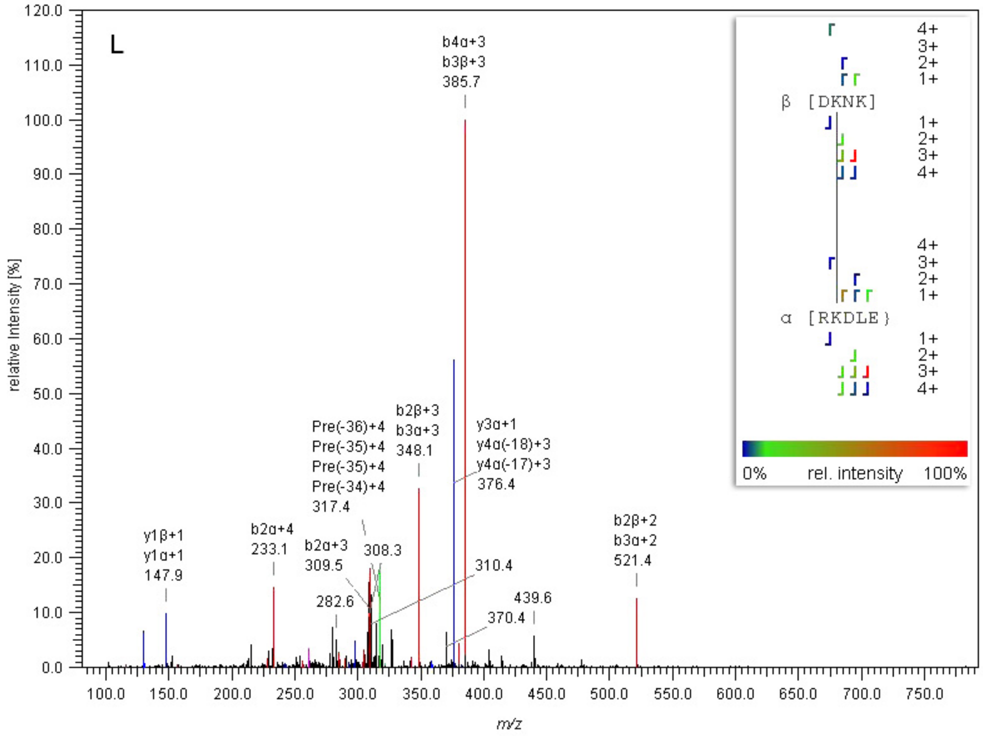

Supplementary Figure 10. Crosslink 10 of table S1. Identified b-ions (red), y-ions (blue) and unidentified signals (black) are indicated in the spectrum. Purple signals represent either b- or y-ions. H: heavy; L: light form of the cross linker

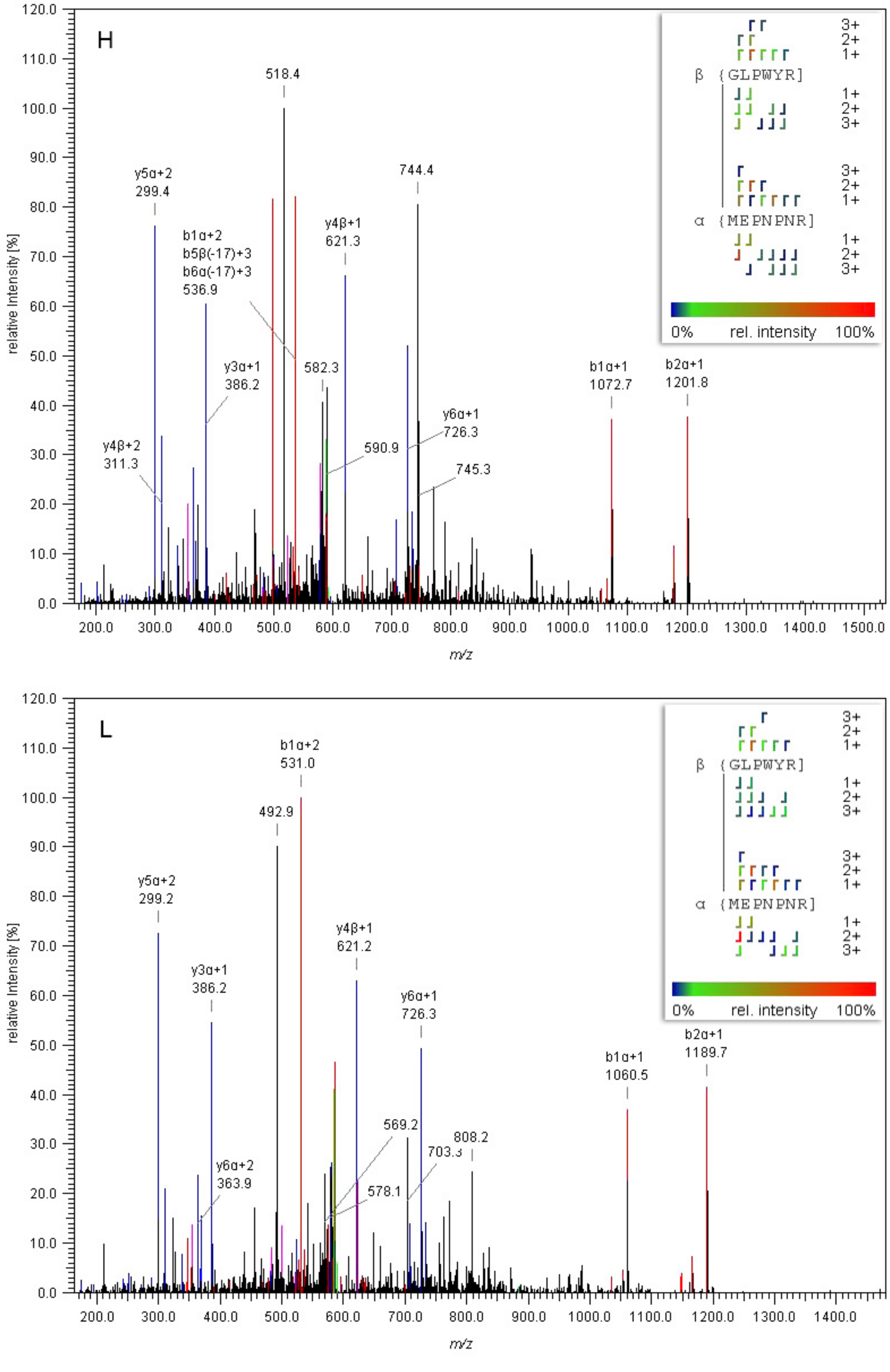

Supplementary Figure 11. Crosslink 11 of table S1. Identified b-ions (red), y-ions (blue) and unidentified signals (black) are indicated in the spectrum. Purple signals represent either b- or y-ions. H: heavy; L: light form of the cross linker

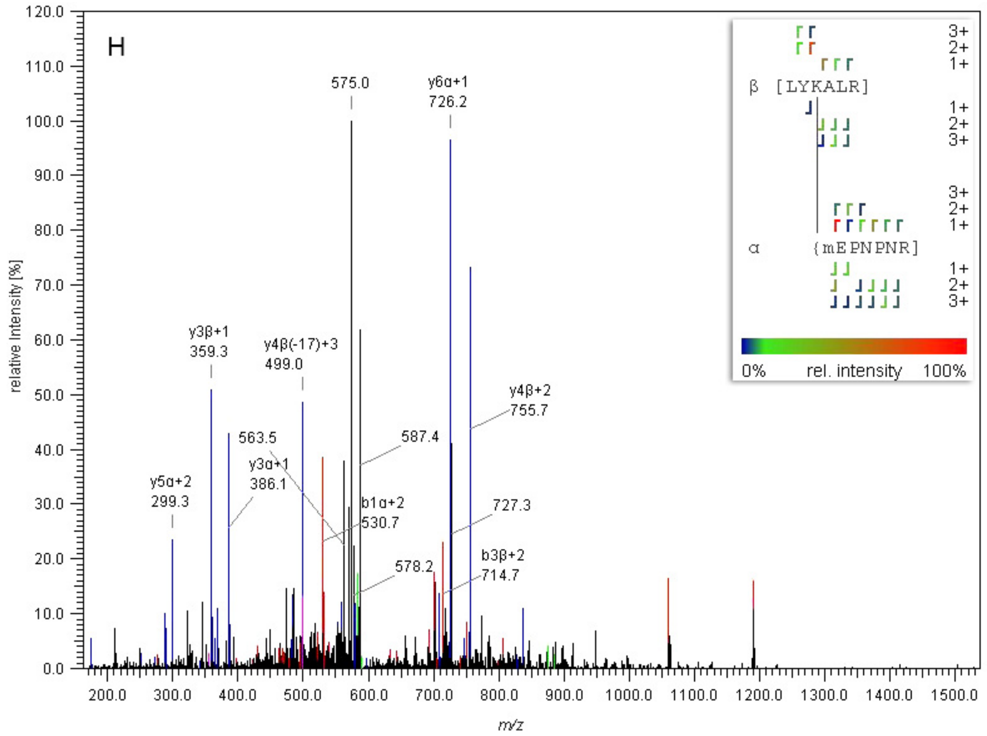

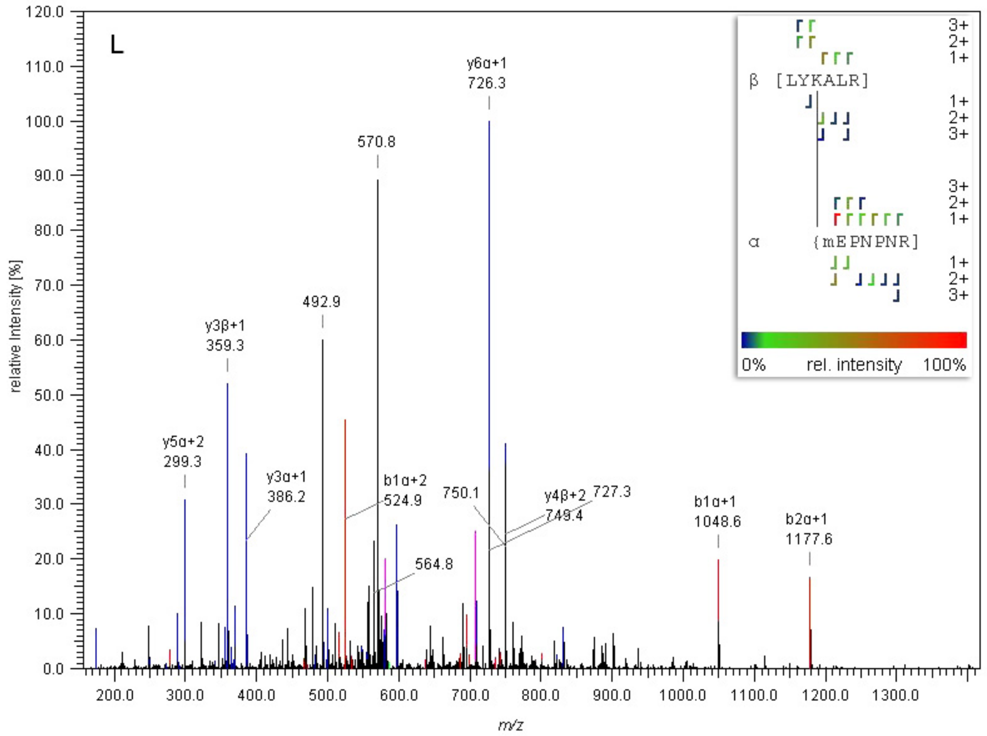

Supplementary Figure 12. Crosslink 12 of table S1. Identified b-ions (red), y-ions (blue) and unidentified signals (black) are indicated in the spectrum. Purple signals represent either b- or y-ions. H: heavy; L: light form of the cross linker

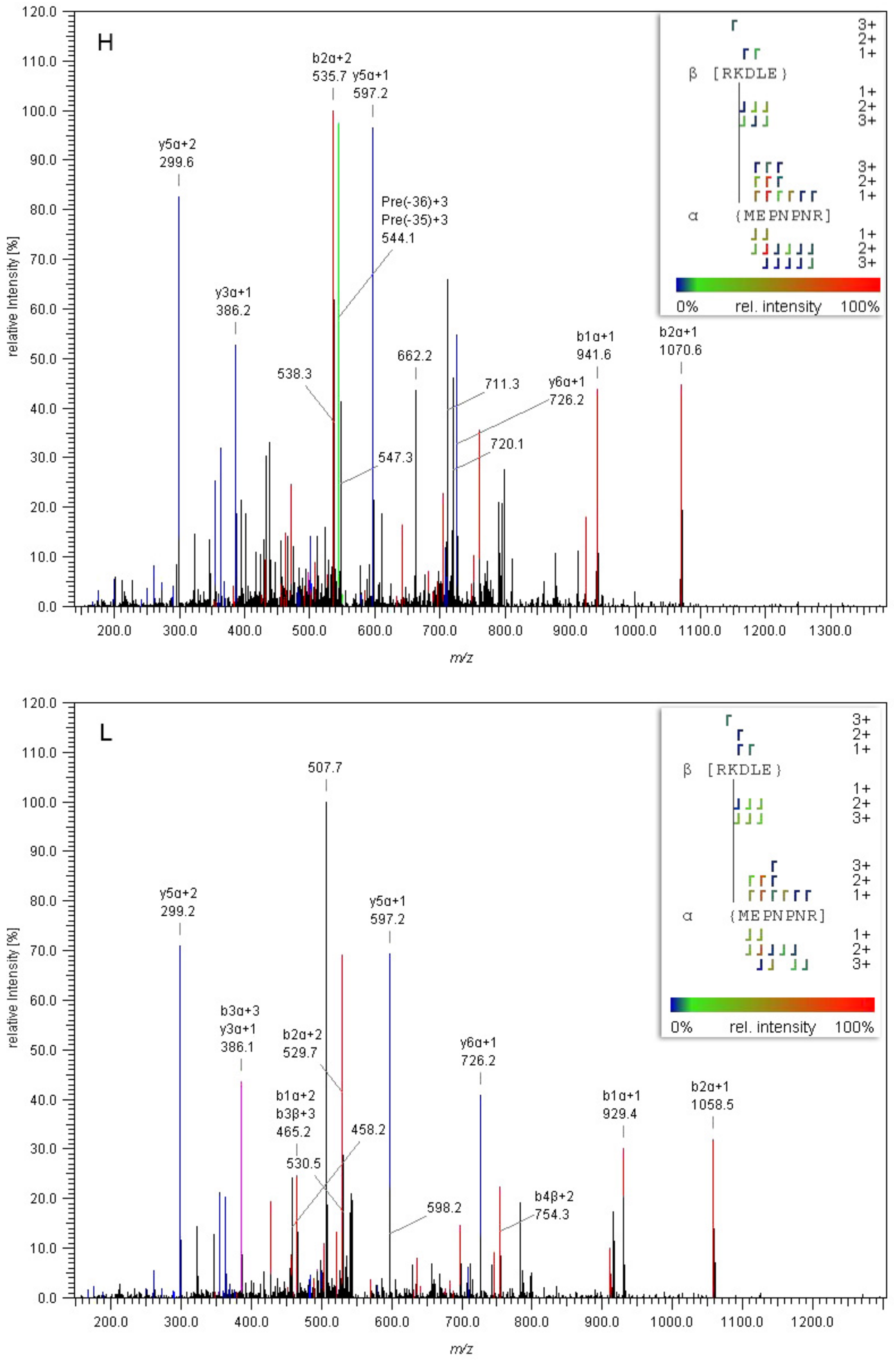

Supplementary Figure 13. Crosslink 13 of table S1. Identified b-ions (red), y-ions (blue) and unidentified signals (black) are indicated in the spectrum. Purple signals represent either b- or y-ions. H: heavy; L: light form of the cross linker

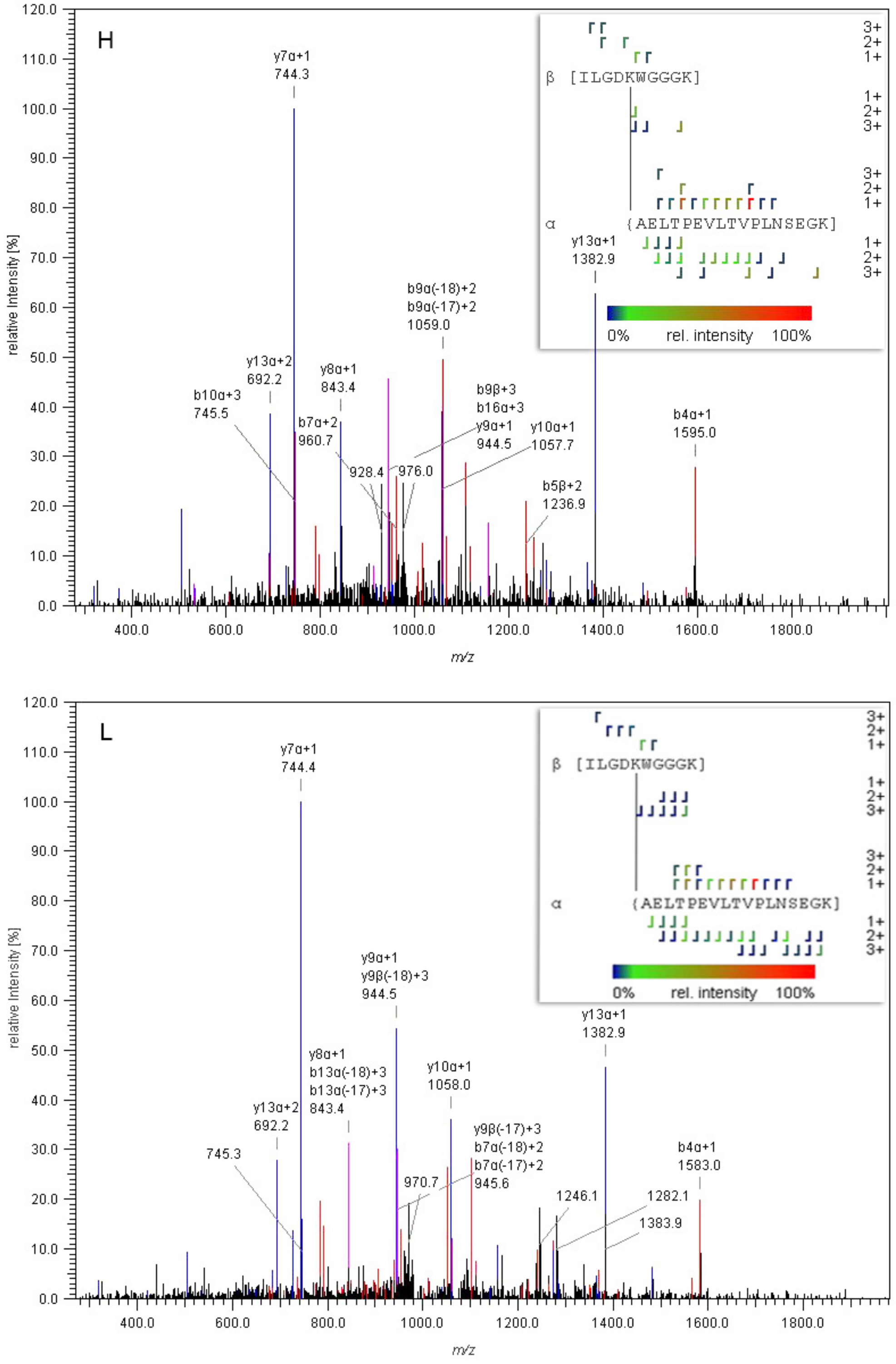

Supplementary Figure 14. Crosslink 14 of table S1. Identified b-ions (red), y-ions (blue) and unidentified signals (black) are indicated in the spectrum. Purple signals represent either b- or y-ions. H: heavy; L: light form of the cross linker

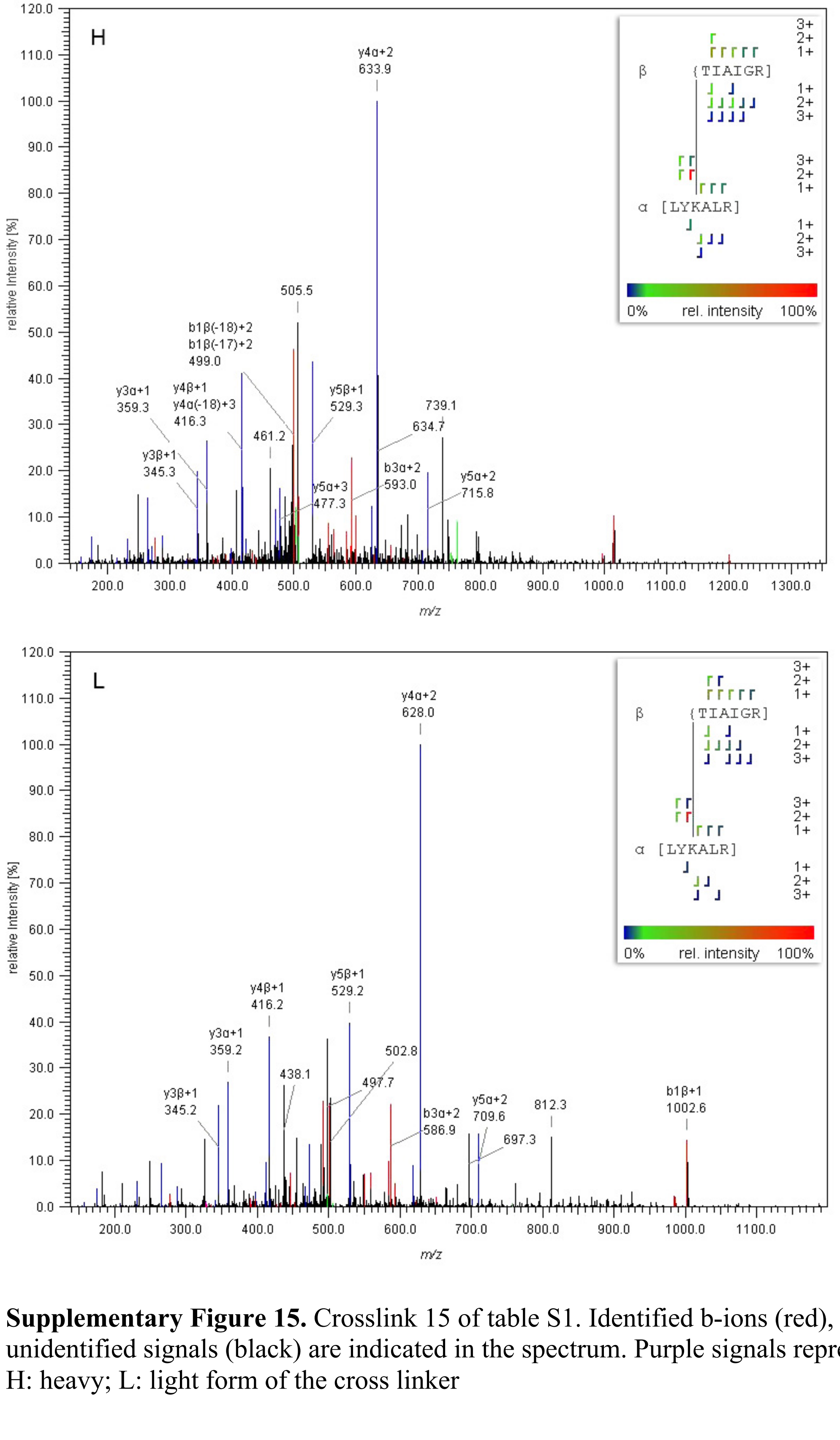

Supplementary Figure 15. Crosslink 15 of table S1. Identified b-ions (red), y-ions (blue) and unidentified signals (black) are indicated in the spectrum. Purple signals represent either b- or y-ions. H: heavy; L: light form of the cross linker

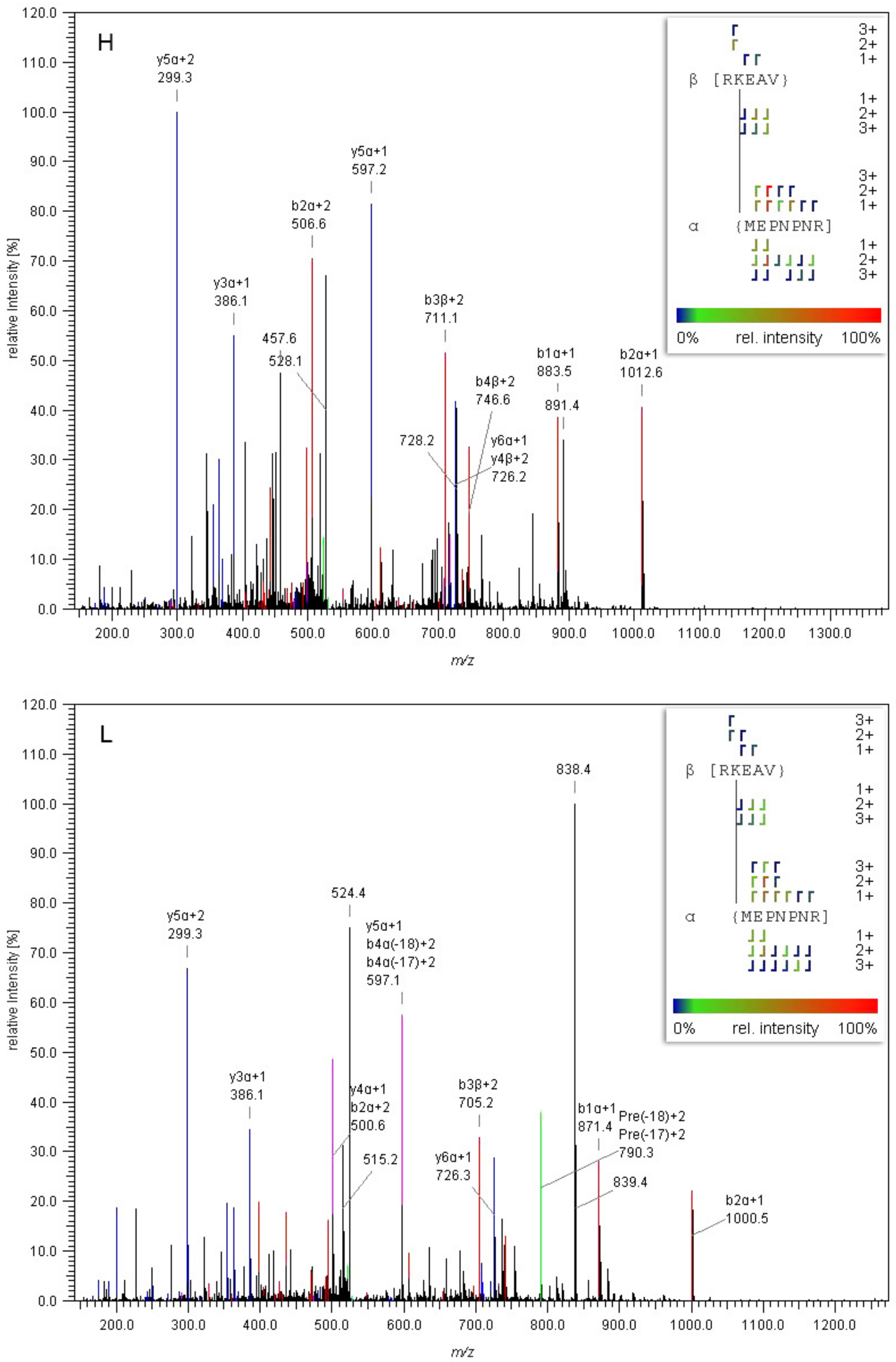

Supplementary Figure 16. Crosslink Ia of table S1. Identified b-ions (red), y-ions (blue) and unidentified signals (black) are indicated in the spectrum. Purple signals represent either b- or y-ions. H: heavy; L: light form of the cross linker

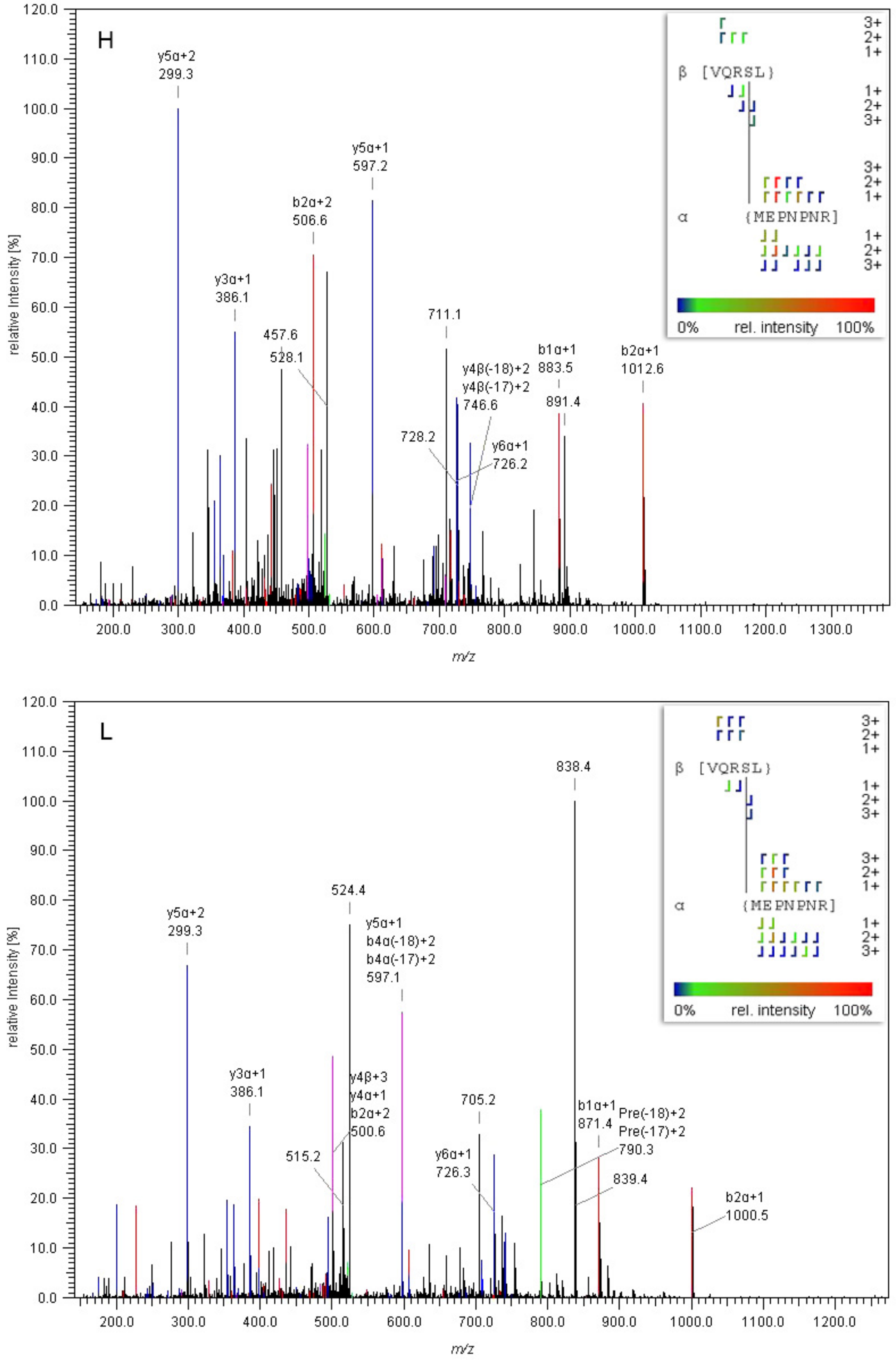

Supplementary Figure 17. Crosslink Ib of table S1. Identified b-ions (red), y-ions (blue) and unidentified signals (black) are indicated in the spectrum. Purple signals represent either b- or y-ions. H: heavy; L: light form of the cross linker

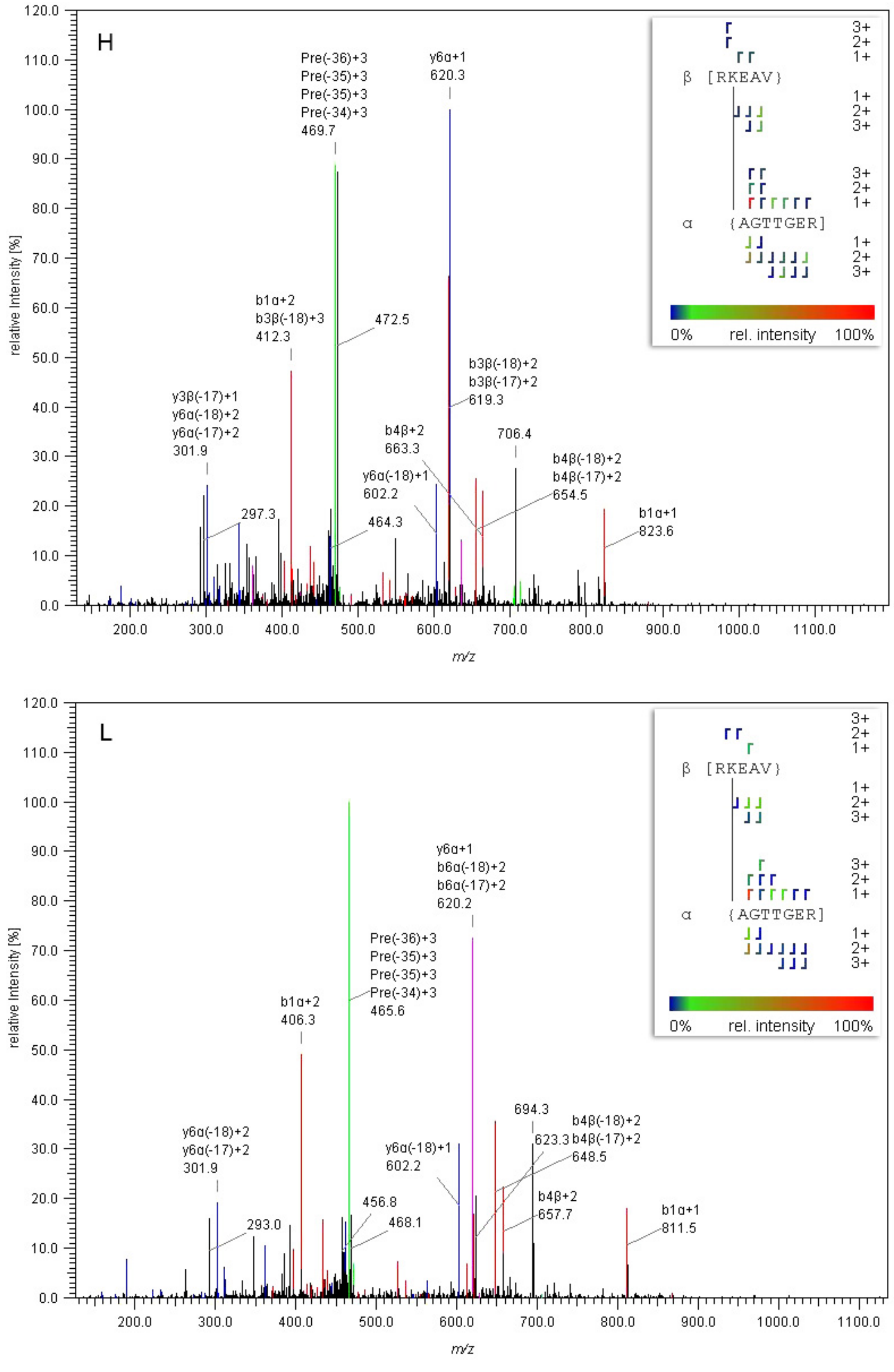

Supplementary Figure 18. Crosslink IIa of table S1. Identified b-ions (red), y-ions (blue) and unidentified signals (black) are indicated in the spectrum. Purple signals represent either b- or y-ions. H: heavy; L: light form of the cross linker

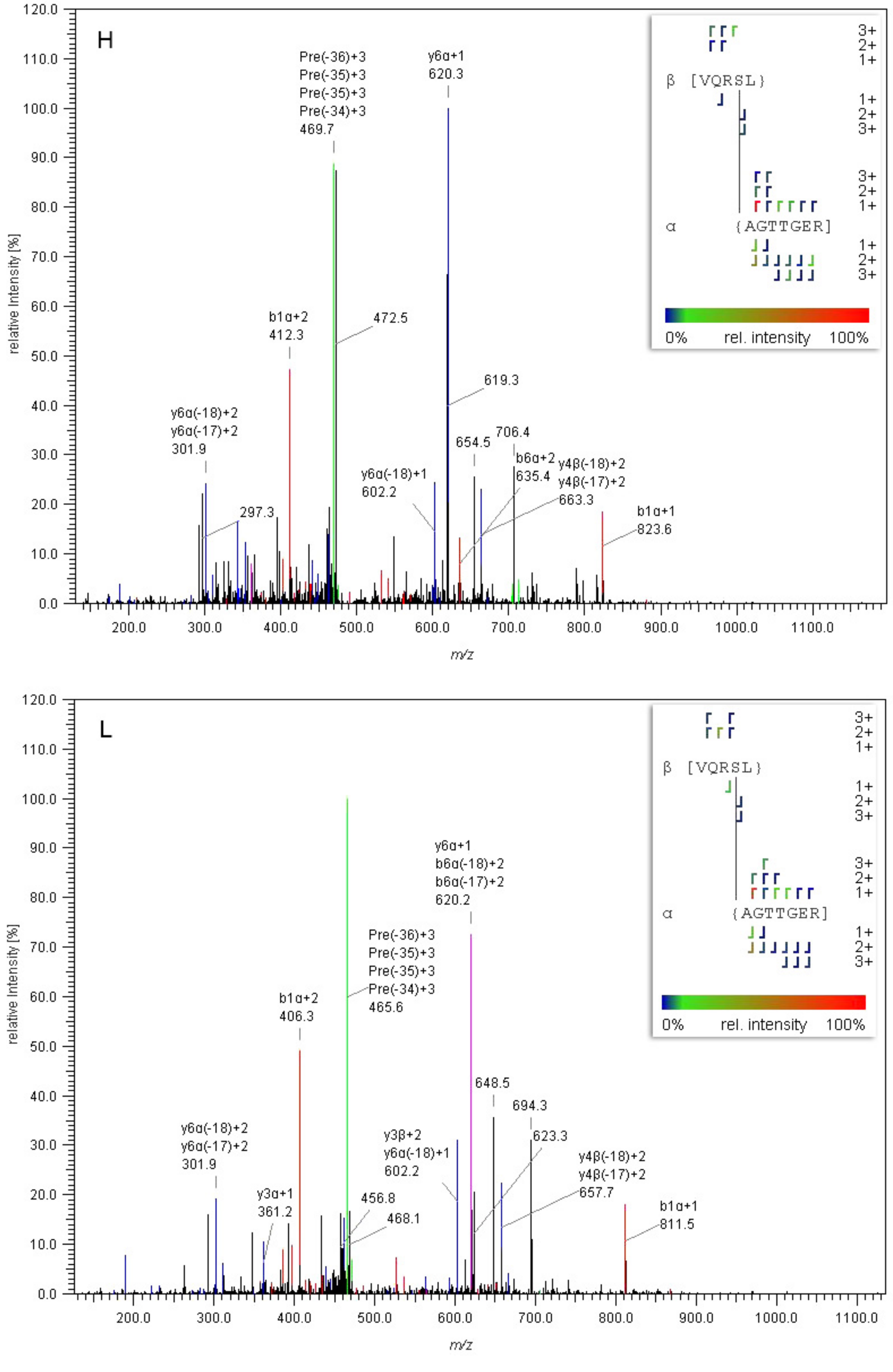

Supplementary Figure 19. Crosslink IIb of table S1. Identified b-ions (red), y-ions (blue) and unidentified signals (black) are indicated in the spectrum. Purple signals represent either b- or y-ions. H: heavy; L: light form of the cross linker

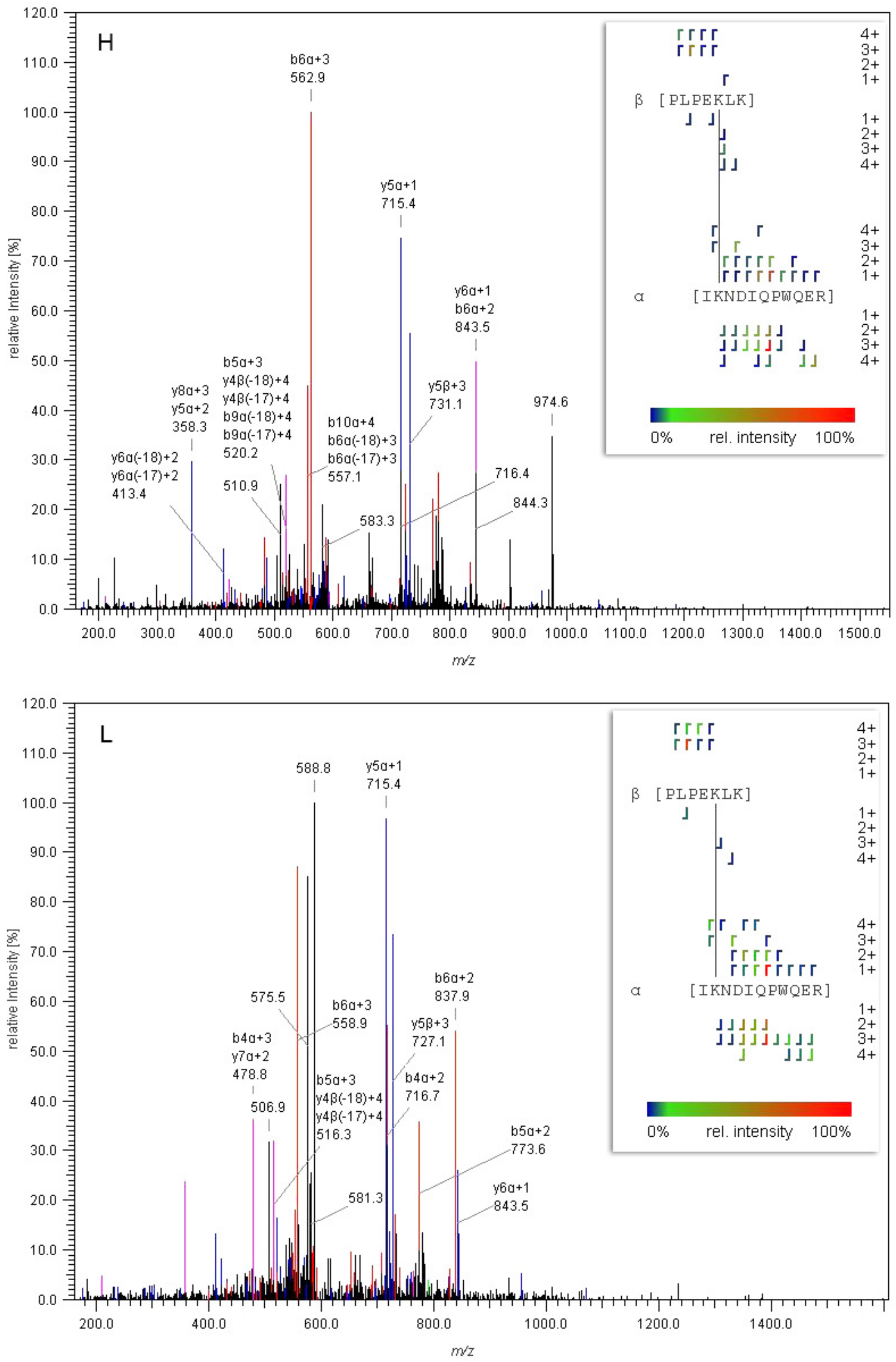

Supplementary Figure 20. Crosslink 1 of table S2. Identified b-ions (red), y-ions (blue) and unidentified signals (black) are indicated in the spectrum. Purple signals represent either b- or y-ions. H: heavy; L: light form of the cross linker

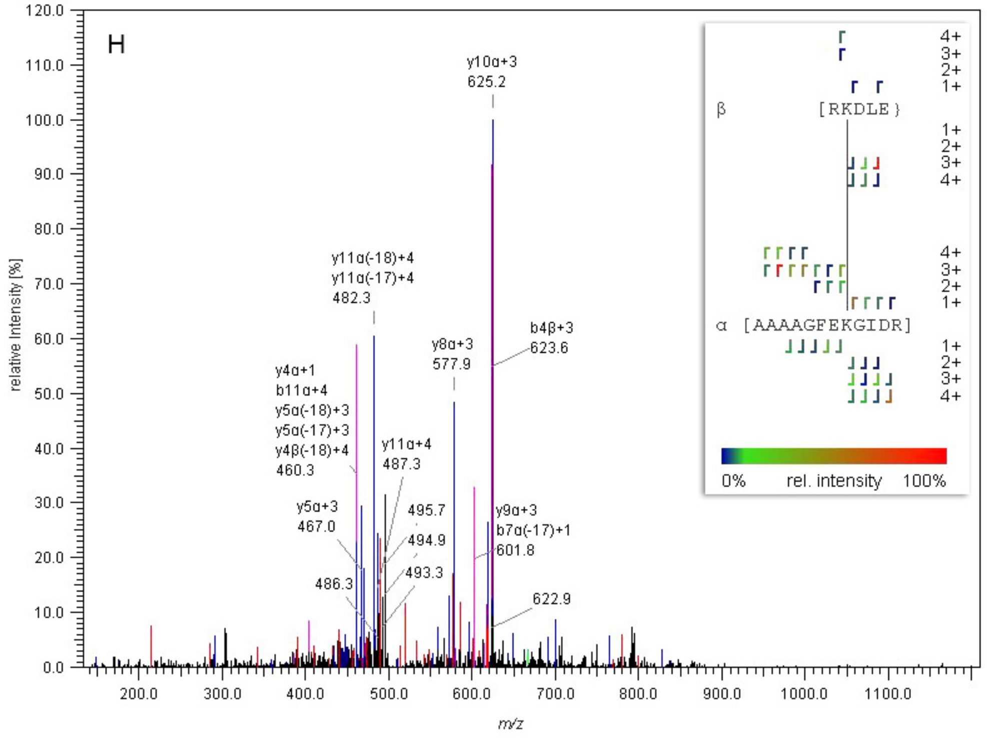

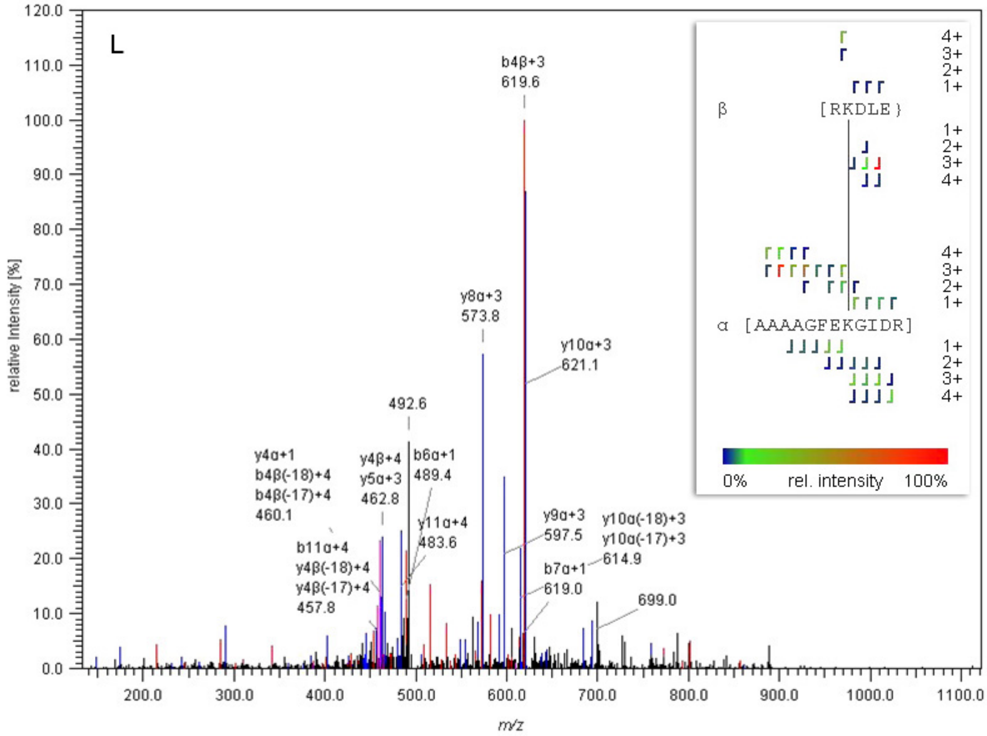

Supplementary Figure 21. Crosslink 2 of table S2. Identified b-ions (red), y-ions (blue) and unidentified signals (black) are indicated in the spectrum. Purple signals represent either b- or y-ions. H: heavy; L: light form of the cross linker

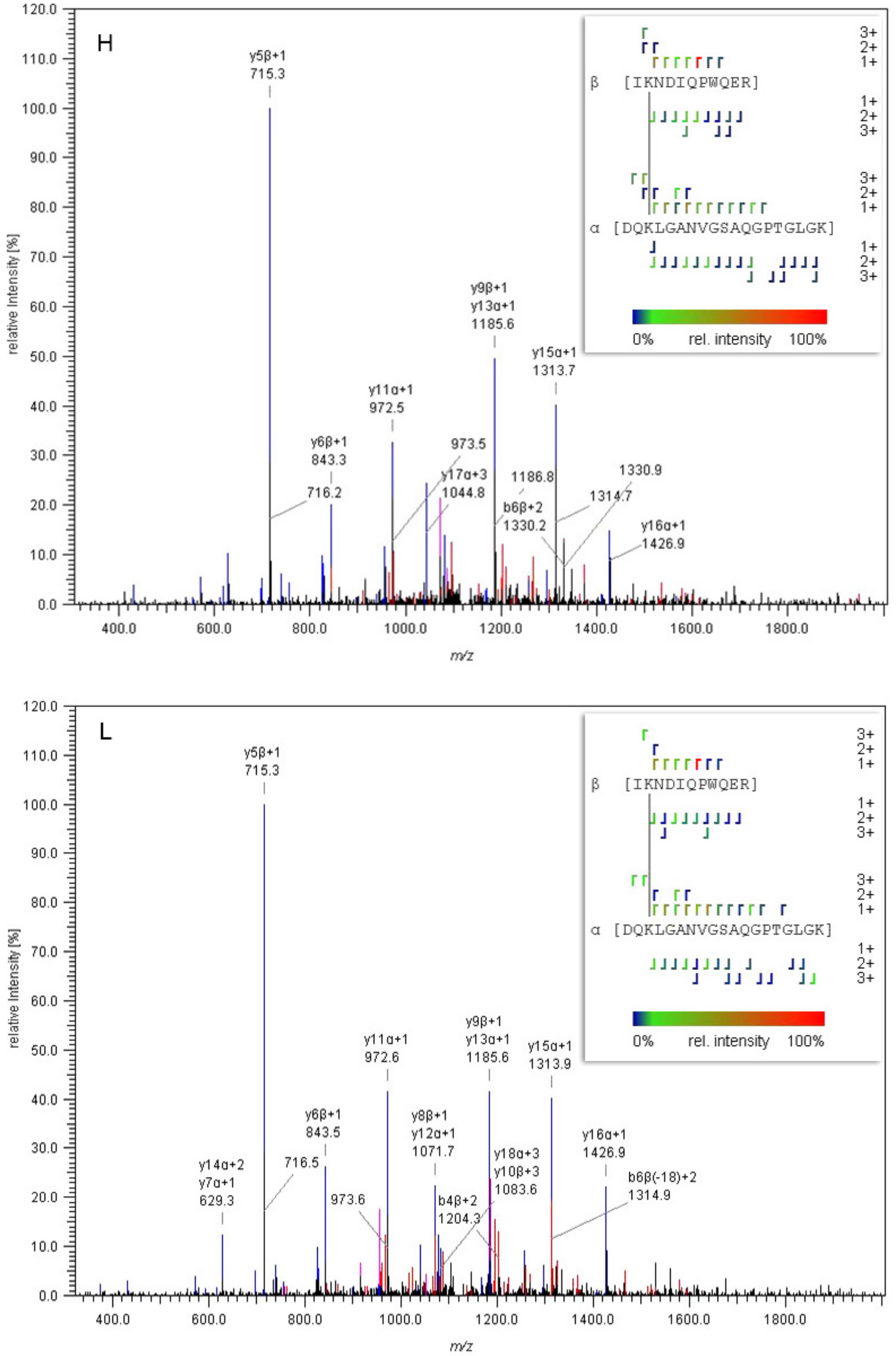

Supplementary Figure 22. Crosslink 3 of table S2. Identified b-ions (red), y-ions (blue) and unidentified signals (black) are indicated in the spectrum. Purple signals represent either b- or y-ions. H: heavy; L: light form of the cross linker

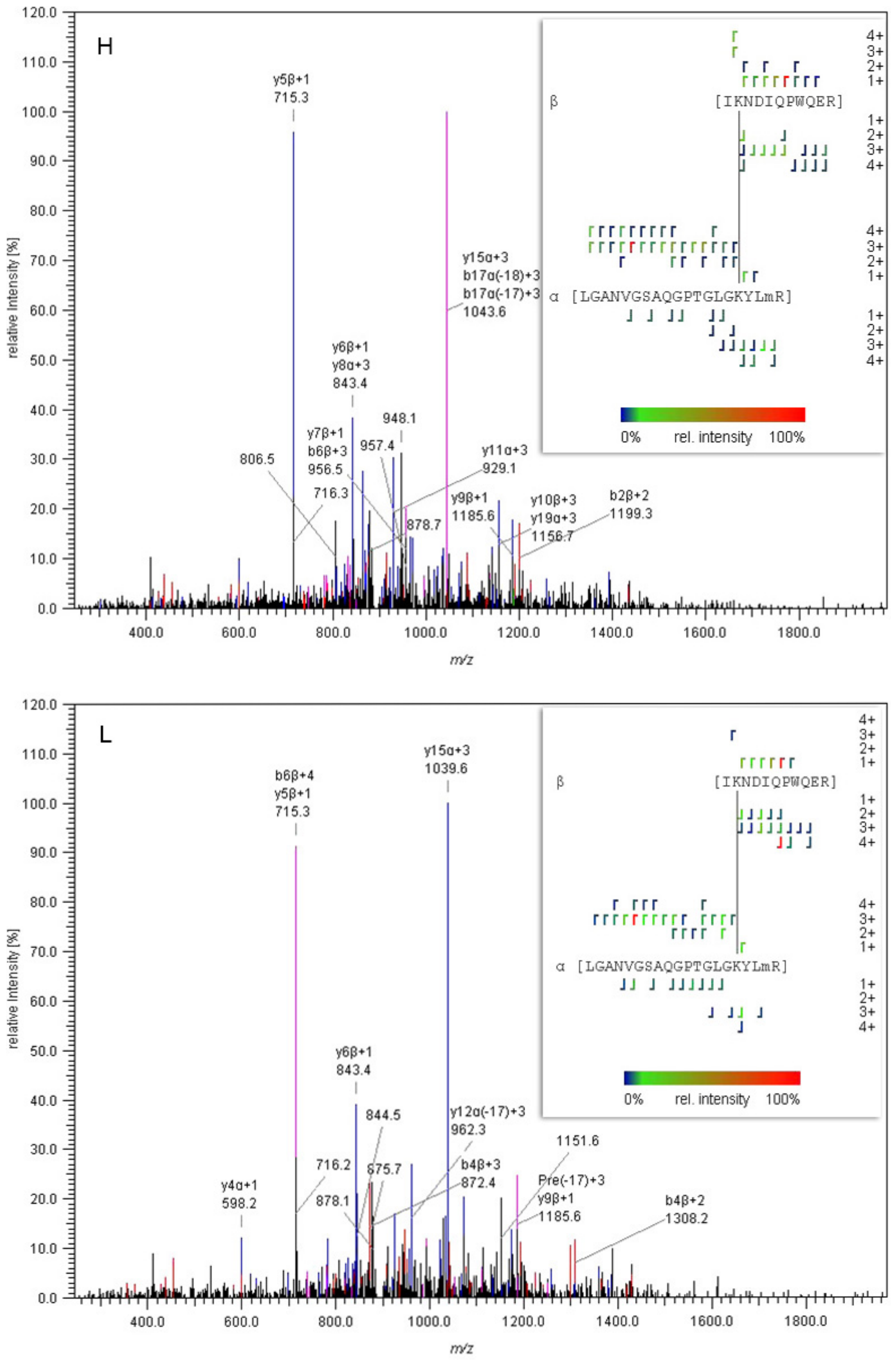

Supplementary Figure 23. Crosslink 4 of table S2. Identified b-ions (red), y-ions (blue) and unidentified signals (black) are indicated in the spectrum. Purple signals represent either b- or y-ions. H: heavy; L: light form of the cross linker

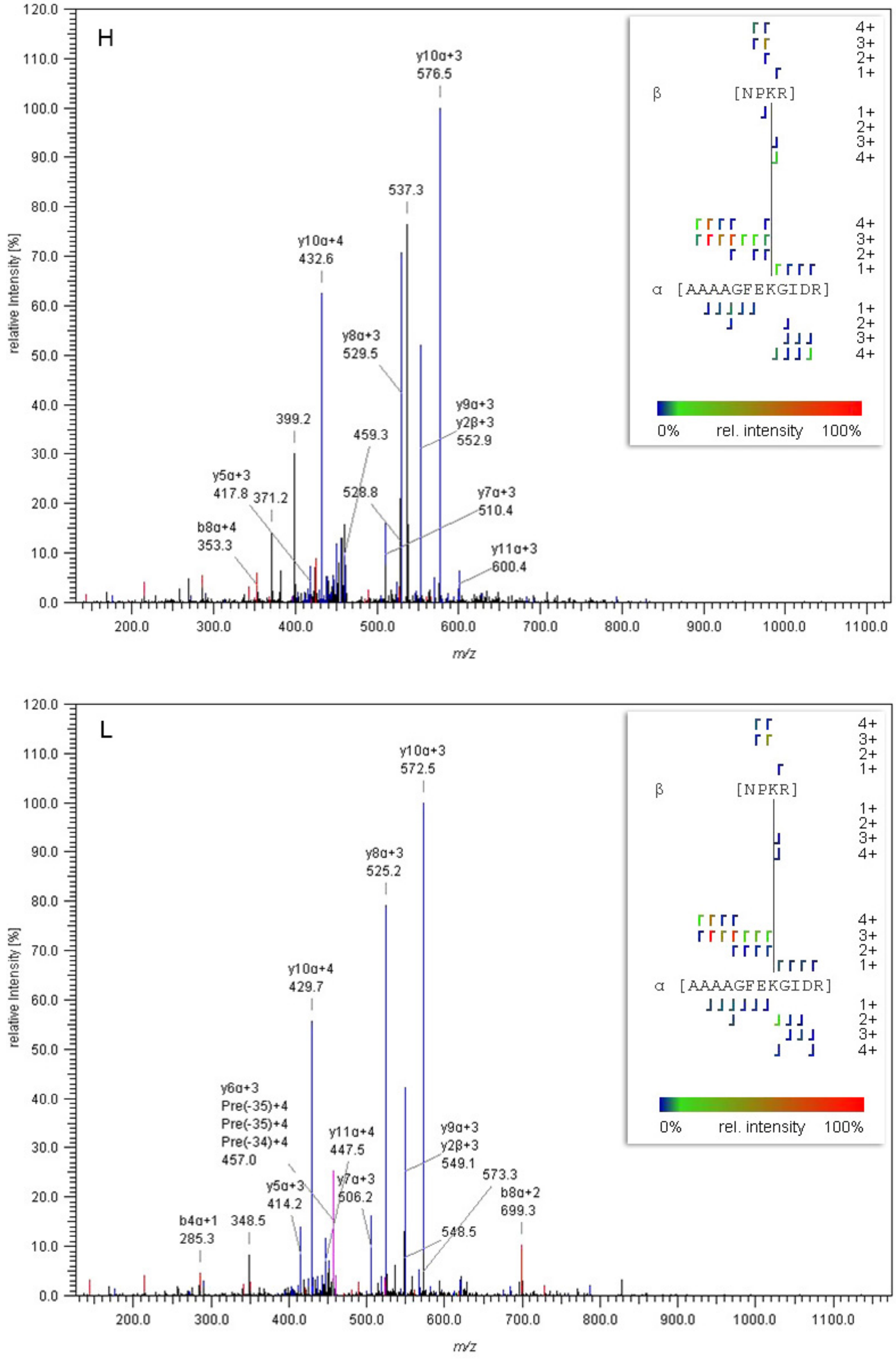

Supplementary Figure 24. Crosslink 5 of table S2. Identified b-ions (red), y-ions (blue) and unidentified signals (black) are indicated in the spectrum. Purple signals represent either b- or y-ions. H: heavy; L: light form of the cross linker

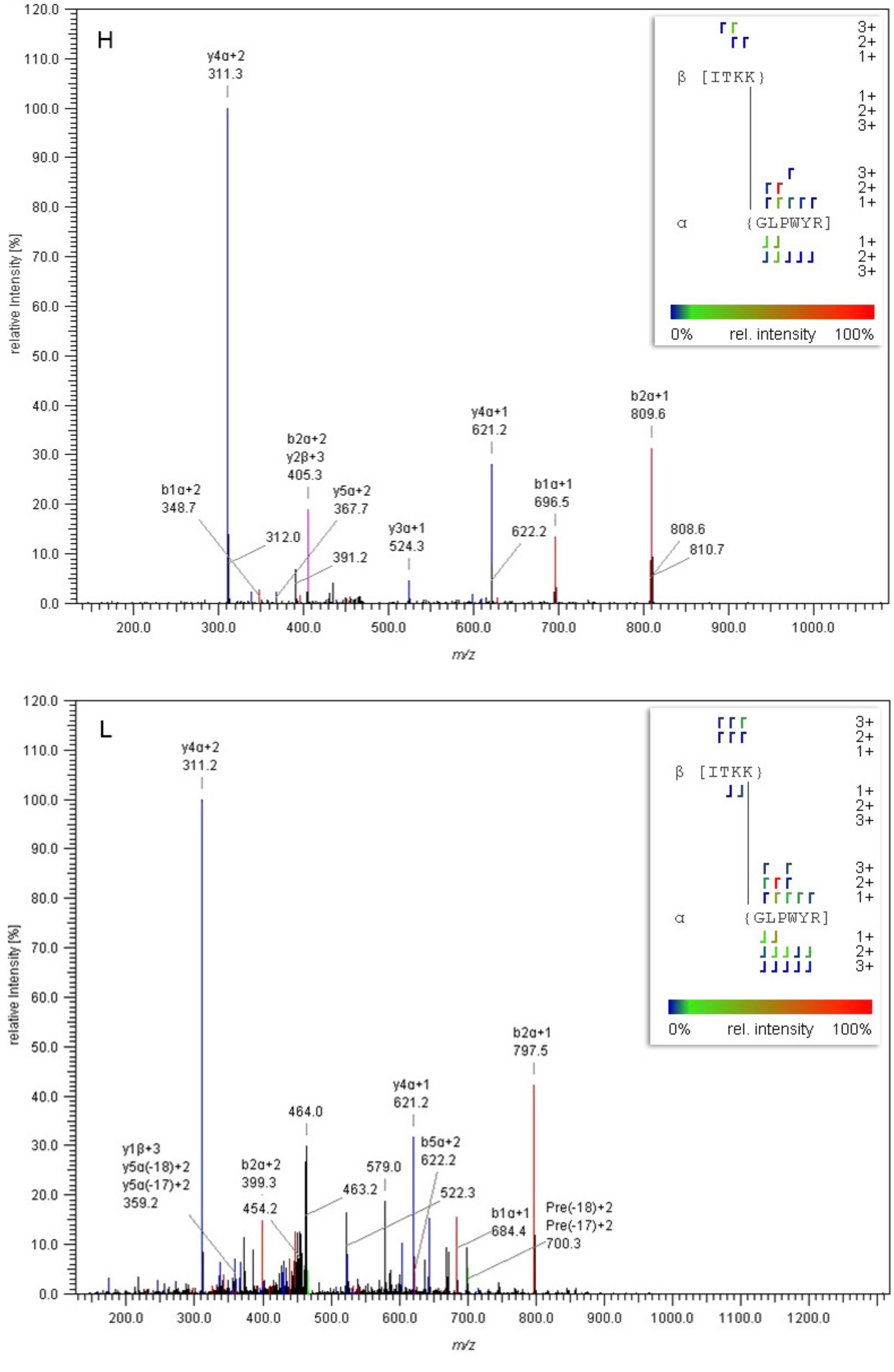

Supplementary Figure 25. Crosslink 6 of table S2. Identified b-ions (red), y-ions (blue) and unidentified signals (black) are indicated in the spectrum. Purple signals represent either b- or y-ions. H: heavy; L: light form of the cross linker

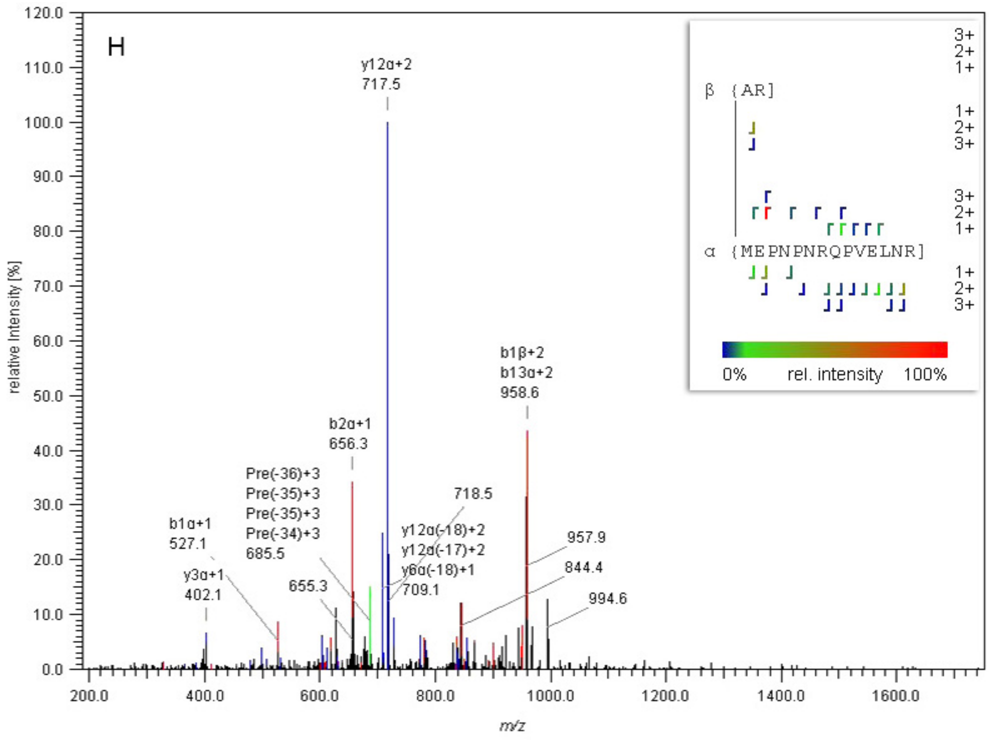

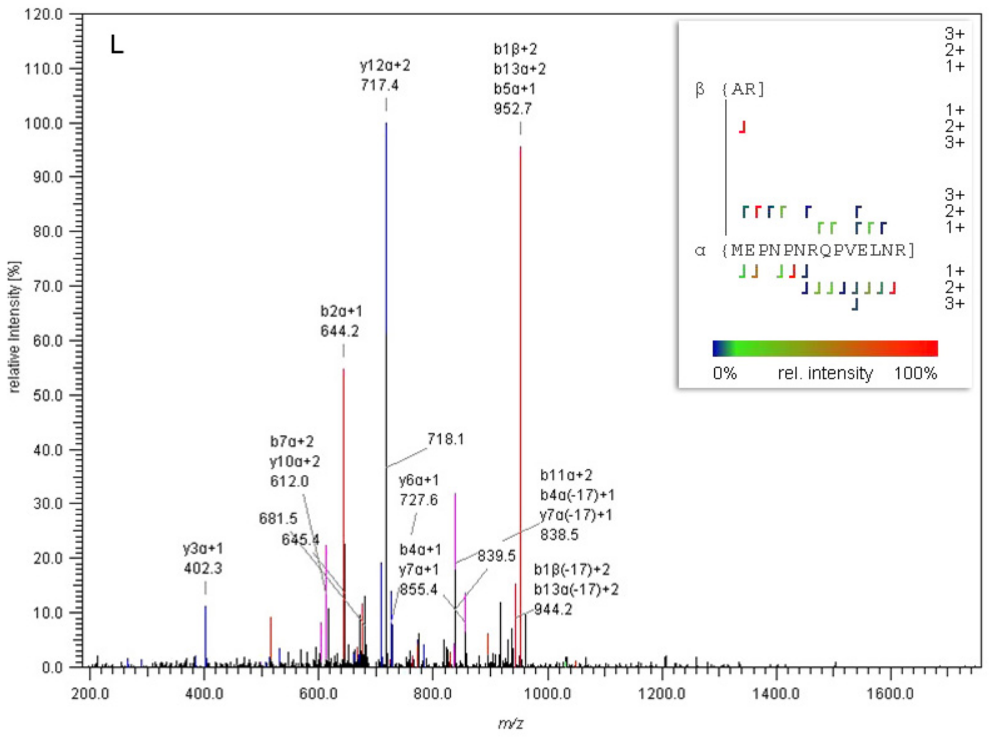

Supplementary Figure 26. Crosslink 7 of table S2. Identified b-ions (red), y-ions (blue) and unidentified signals (black) are indicated in the spectrum. Purple signals represent either b- or y-ions. H: heavy; L: light form of the cross linker

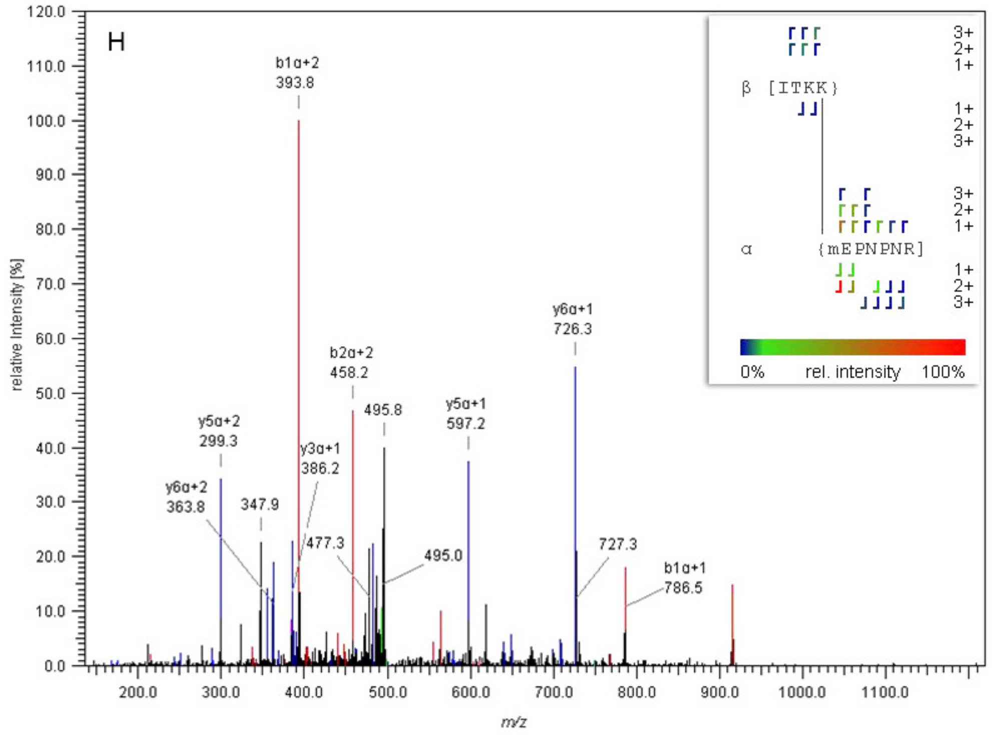

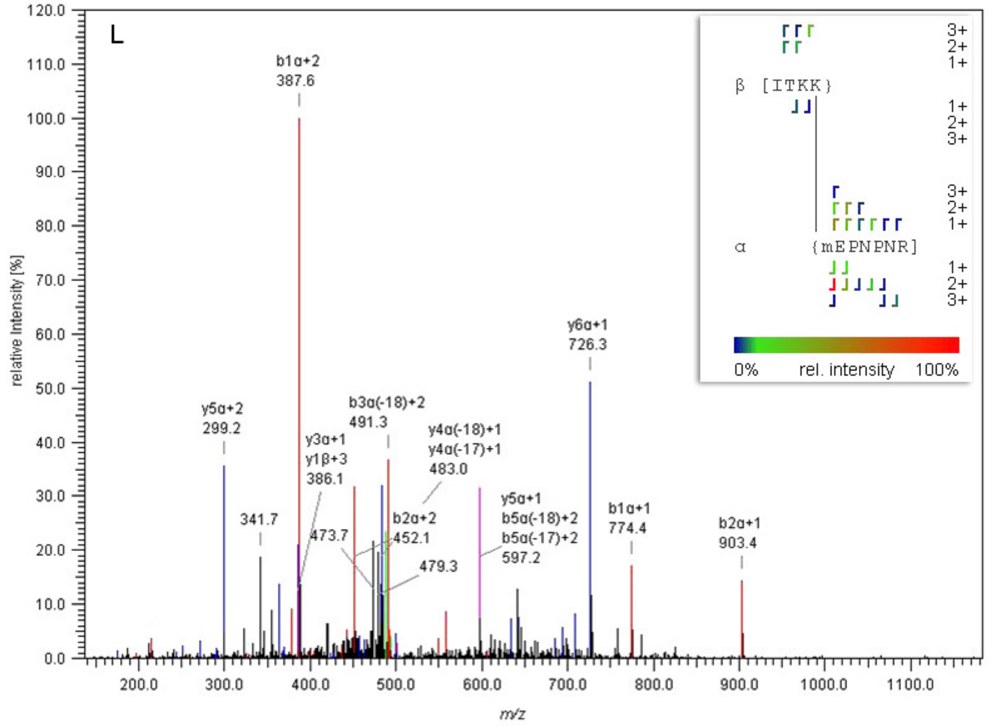

Supplementary Figure 27. Crosslink 8 of table S2. Identified b-ions (red), y-ions (blue) and unidentified signals (black) are indicated in the spectrum. Purple signals represent either b- or y-ions. H: heavy; L: light form of the cross linker

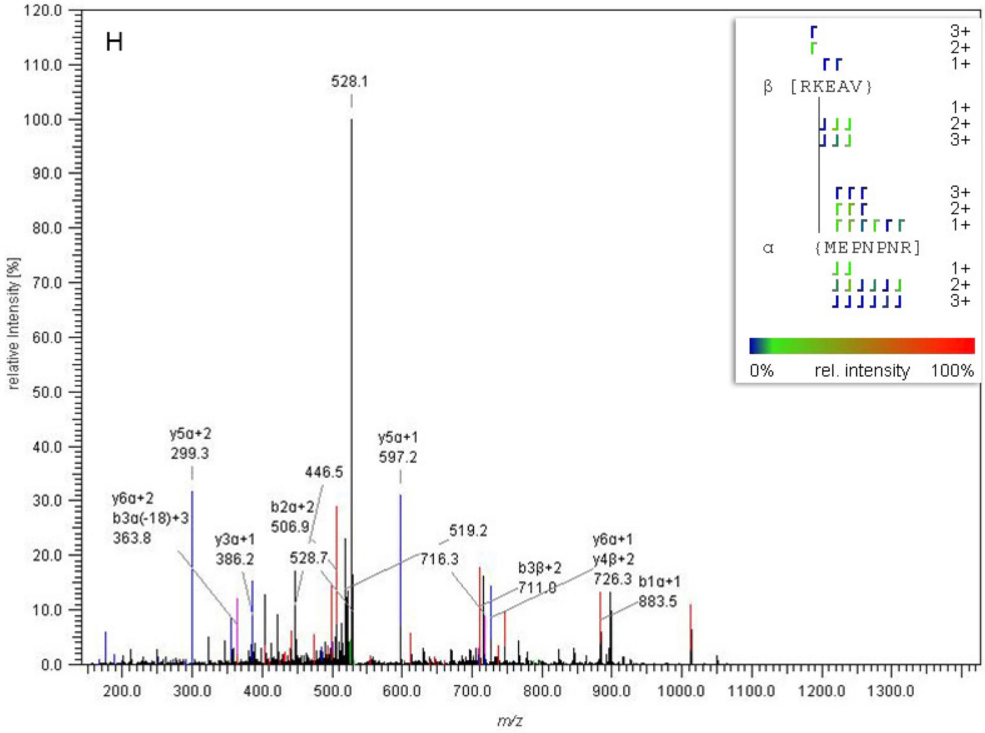

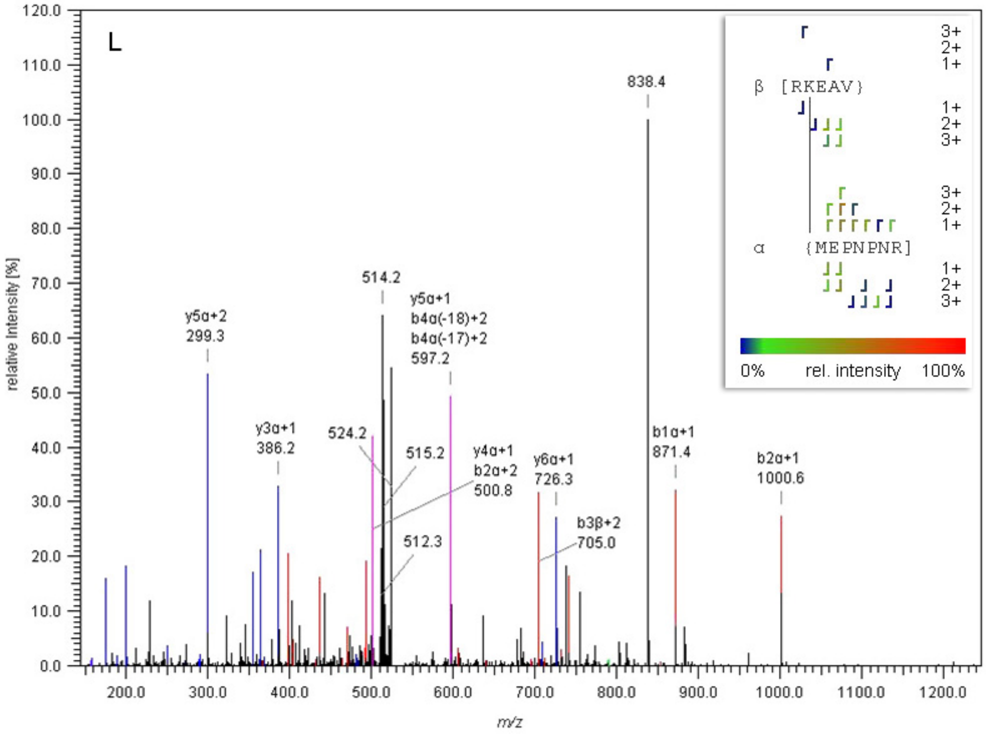

Supplementary Figure 28. Crosslink Ia of table S2. Identified b-ions (red), y-ions (blue) and unidentified signals (black) are indicated in the spectrum. Purple signals represent either b- or y-ions. H: heavy; L: light form of the cross linker

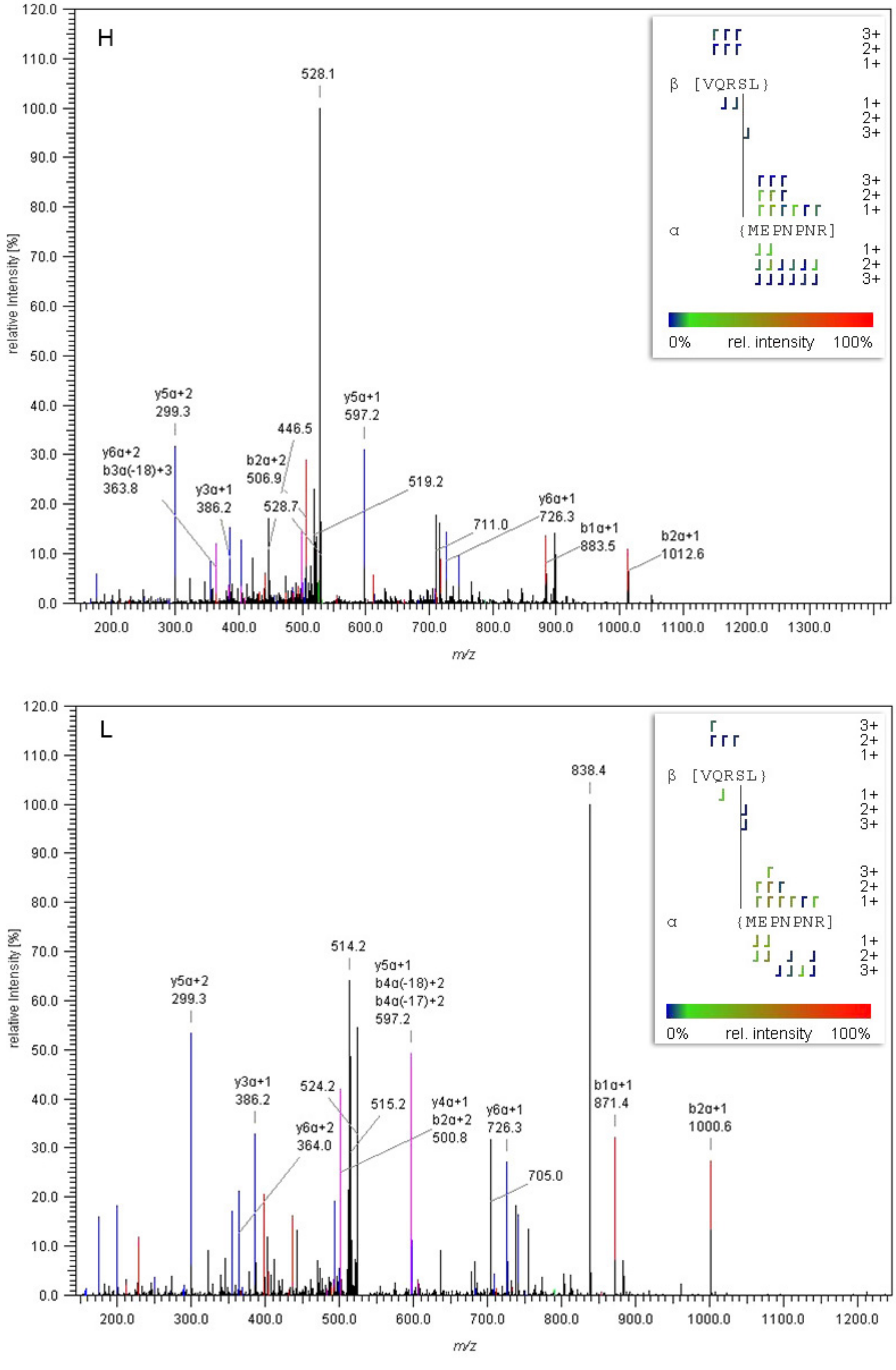

Supplementary Figure 29. Crosslink Ib of table S2. Identified b-ions (red), y-ions (blue) and unidentified signals (black) are indicated in the spectrum. Purple signals represent either b- or y-ions. H: heavy; L: light form of the cross linker
